# Parameter identification of a delayed infinite-dimensional heat-exchanger process based on relay feedback and root loci analysis

**DOI:** 10.1038/s41598-022-13182-5

**Published:** 2022-06-03

**Authors:** Libor Pekař, Mengjie Song, Subhransu Padhee, Petr Dostálek, František Zezulka

**Affiliations:** 1 Department of Automation and Control Engineering, Faculty of Applied Informatics, Tomas Bata University in Zlín, nám. T. G. Masaryka 5555, 760 01 Zlín, Czech Republic; 2 Department of Technical Studies, College of Polytechnics Jihlava, Tolstého 1556/16, 586 01 Jihlava, Czech Republic; 3 Department of Energy and Power Engineering, School of Mechanical Engineering, Beijing Institute of Technology, Engine East Building 125, Beijing, 100081 China; 4 Department of Electrical and Electronics Engineering, Sambalpur University Institute of Information Technology, Burla, Sambalpur, 769018 India

**Keywords:** Chemical engineering, Electrical and electronic engineering, Thermoelectric devices and materials, Process chemistry, Computational science

## Abstract

The focus of this contribution is twofold. The first part aims at the rigorous and complete analysis of pole loci of a simple delayed model, the characteristic function of which is represented by a quasi-polynomial with a non-delay and a delay parameter. The derived spectrum constitutes an infinite set, making it a suitable and simple-enough representative of even high-order process dynamics. The second part intends to apply the simple infinite-dimensional model for relay-based parameter identification of a more complex model of a heating–cooling process with heat exchangers. Processes of this type and construction are widely used in industry. The identification procedure has two substantial steps. The first one adopts the simple model with a low computational effort using the saturated relay that provides a more accurate estimation than the standard on/off test. Then, this result is transformed to the estimation of the initial characteristic equation parameters of the complex infinite-dimensional heat-exchanger model using the exact dominant-pole-loci assignment. The benefit of this technique is that multiple model parameters can be estimated under a single relay test. The second step attempts to estimate the remaining model parameters by various numerical optimization techniques and also to enhance all model parameters via the Autotune Variation Plus relay experiment for comparison. Although the obtained unordinary time and frequency domain responses may yield satisfactory results for control tasks, the identified model parameters may not reflect the actual values of process physical quantities.

## Introduction

It is a well-known fact that dozens of industrial processes, including chemical ones, as well as social, economic, and other everyday systems are affected by latencies and delays^1–6^. Delays appear mainly due to mass, energy, and data transportation in the process and network interconnections, and their existence is closely related to distributed parameter systems. In modern discrete-time control systems, delays also arise from the human–machine interaction and signal sampling and processing^7^. As complex systems include internal feedback loops, internal delays must be considered along with the input–output ones; nevertheless, internal delays are often ignored when process modeling. However, such an approach can be unreasoning as the solution of partial differential equations (PDEs)—representing the reign of many industrial process models—often results in functions with lumped and distributed delays ^8–10^.

On the other hand, time-delay models (TDMs) may be very good estimators of some systems and processes dynamics, even if any significant physical delay is not supposed to appear in the process. TDMs have the form of functional differential equations, or more specifically, delay differential equations (DDEs), instead of PDEs. Even a simple TDM can express the dynamics of a high-order non-delay model^11,12^ with sufficient accuracy for control design. However, these models are infinite-dimensional because of the transcendental form of the characteristic equation (CE)^13^. All infinitely many solutions of the CE constitute the TDM spectrum of characteristic values (or poles). The pole loci most significantly determine the dynamic and stability properties of the model^14^. The infinite nature of the TDM spectrum yields its advantages and disadvantages when estimating the actual system dynamics. The dominant (i.e., usually the rightmost) subset of poles can match the pole loci of a high order system; however, one must be careful about other (uncontrolled) TDM poles, especially of high-frequency ones.

Various methods and techniques for the TDM spectrum analysis and its pole loci estimation exist; see a survey by Pekař and Gao^15^. We do let provide the reader with just a few. If TDMs have the so-called commensurate delays solely, pole loci can be determined analytically via the Lambert W function^16^. However, this is the only exact method besides the direct solution of the CE via the analysis of the distribution of the roots of the corresponding CE in the frequency domain, see, e.g., the work by Amrane et al.^17^. The family of numerical methods includes a wide variety of approaches and techniques that are based on, e.g., the mapping of real and imaginary parts of CEs solutions^18^, bifurcation analysis of DDEs^19^, full discretization of TDMs^3^, continuation property of TDMs pole loci^7^ or on structural properties of a class of functional Vandermonde matrices^20^.

TDMs pole loci analysis is closely related to the inverse problem of the (partial) spectrum assignment, i.e., the determination of the model parameter (or even delay) values so that a subset of the characteristic values match the prescribed positions while other poles are sufficiently away from the chosen loci. There, again, exist several computational methods as well as principles of how to select the desired loci. The direct root assignment is essentially the most straightforward technique that gives rise to the solution of a set of algebraic (linear or nonlinear) equations^21^, the dimension of which is given by the number of assigned roots, their multiplicity and complexity. Its computational simplicity is, however, ransomed by a danger of a possible existence of model poles located right from the prescribed ones, which yields a problem of the poles’ dominancy. Only a few tools ensure that the desired TDMs poles are dominant, e.g., a modified Nyquist stability criterion can be applied^22^, yet it is based on a graphical trial-and-reset procedure. Recently, several ad-hoc results for single^17^ and multiple real^20,23^ prescribed poles or even single a complex conjugate pair^24^ guarantying their dominancy have been derived; however, they usually levy large computational burden. Alternatively, the root dominance can be a posteriori checked using the argument principle (i.e., the Mikhailov curve based) approach^25^ or via the solution of a special convolution integral^26^, which requires an advanced mathematical effort as well. Whenever the direct assignment is not satisfactory, a numerical spectrum optimization can be made, e.g., by the quasi-continuous shifting of the roots^27,28^ or using its combination with the minimization of a specific fitness function reflecting the remaining spectrum, robustness issues, etc.^29–31^. Unfortunately, a non-convex optimization problem must be solved in many cases, see, e.g.,^32^ and references therein.

The use of relay in the feedback control system represents a favorite system parameters’ identification and automatic controller tuning framework that have received a great deal of attention since the pioneering work by Åstrom and Hägglund^33^, where the ideal on/off relay was used to generate sustained (ultimate) oscillations. This parameter estimation framework enables to prevent the process output from drifting too far away from the reference signal (setpoint), which is required for many industrial processes. During recent decades, a multitude of derived techniques and methods have been developed^34,35^ that have found a great favor of practitioners, especially in chemical and process engineering^36–41^.

Three families of approaches to evaluate unknown model parameters^34^ exist. Namely, using a describing function (DF) represents the most common approach^33,38,42^. Roughly speaking, this function is a linear approximation (usually based on the Fourier series expansion) of the nonlinear relay behavior. Second, the curve fitting approach attempts to fit the feedback response in the time domain based on an analytic formulation of the response^43–46^. As third, the frequency fitting does the same yet in the frequency domain, which corresponds to the seeking of multiple frequency points, besides the ultimate case^47–49^.

It is known that a sufficiently accurate process model can reduce errors in controller tuning^50^. The ultimate gains obtained from the standard (on/off) relay feedback oscillation amplitudes can have errors of over 15%^51^. Besides, Jeon et al.^52^ pointed out that model parameters obtained from the sustained relay oscillations can be insufficient if there is a mismatch in the model order and process dynamics, which gives rise to the need for more complex models. However, as the original method suffers from significant errors in model parameters’ estimation and only one (critical) point of the frequency characteristics (i.e., two model parameters) can be obtained from the test, researchers have developed methods to fit the parameters more precisely and/or to search for more frequency response points under one or more relay tests.

Regarding the former group of methods, improved accuracy can be obtained by compensating for the phase lag caused by the relay module^53^—which is suitable for higher-order processes or those with large input–output delay, by using a biased relay^54^, a relay with two-band hysteresis to reduce the oscillation frequencies^55^ or relays with multiple switching^56,57^ that prune the relay oscillation harmonics and the effect of the input nonlinearity. Unfortunately, the reduction of the oscillation frequencies increases the experimental times. Other techniques, e.g., attempt to obtain as sinus-like relay output as possible by using a relay with saturation^35,58,59^, to reduce the effect of noises and disturbances^50,60^, to apply the so-called area methods that integrate specifically modified time responses^61^ or to use asymmetrical limit cycle^62^ or a shape factor^63^. However, most methods suffer from the sensitivity to plant–model structural mismatch^58^.

The simplest approach how to obtain more frequency points is to perform more than one relay test using an additional integrator^64,65^, via the parasitic relay^66^, a relay with hysteresis^67^ or the biased relay^42^ that alter the DF^68^. Li et al.^69^ proposed a well-applicable method called the Autotune Variation (ATV) that introduces an artificial delay in an additional test following the standard relay experiment. The technique was further improved by Kim^70^, Marchetti and Scali^38^, and Scali et al.^71^. These methods are popular among practitioners due to their computational simplicity. However, multiple experiments may be time consumptive; therefore, researchers have attempted to gain information about more frequency points or other system dynamic features under a single relay test. Even a purposefully induced asymmetry can be used to determine additional frequency response points without performing additional relay feedback tests^72^. Nevertheless, the given asymmetry may yield the termination of oscillations, which fails the relay test. Numerous other techniques exist, such as the shifting method^51,73,74^, the use of relay transient^48,54,75^ or the computation of weighting moments^47,61^.

Most of the linear process models used in literature when applying some of the relay-based identification experiments include input–output delays within First-Order plus Dead Time (FOPDT) or Second-Order plus Dead Time SOPDT models, either stable^44–46,50,55,63,69,76^ or unstable^43,44,51,77–81^ ones. The models are represented simply by a series of a delay-free low-order submodel and the delay element. However, FOPDT models can suffer from poor performances for some low-order processes with fast parasitic dynamics^61^. Besides, the delay value is hardly ever considered as significant or dominant, even if such an assumption is often unreasonable in practice^82^.

Surprisingly, not many results concern internal delays incorporated in TDMs. Parameter estimate of TDMs is more complicated than parameter estimate of standard models^47^. The pioneering work in this field was presented by Vyhlídal and Zítek^12^. The authors comprised a first-order-derivative model with one internal delay parameter within the standard relay test^33^. We do let call this TDM the Simple First-Order one (SFOTDM) for further presentation. The identified SFOTDM was further used for internal model control design. These results were taken as a starting point for parameter estimation of a TDM of the continuous stirred-tank reactor. The parameter values are further enhanced by solving nonlinear objective functions governed by the difference of the model and measured responses in the time domain via an—optimization algorithm. Nevertheless, a relay was not used in the proposed design; the authors only referred to the awkward need to determine the input–output delay value before the relay test. Pekař and Prokop^83^ compared the use of the saturated relay^42^ and the limit-cycle evaluation using the exponential decaying followed by the discrete-time Fourier transform^54,66^. The authors considered a first-order-derivative TDM that included three non-delay parameters, one internal delay parameter, and the dead time as the model of a circuit system with heat exchangers^84^. An artificial delay was used for the additional relay test. In^47^, the method of moments^64^ was applied to the serial combination of the SFOTDM and a low-pass filter represented by the delay-free first-order submodel^85^, and to its high-order generalization that, however, does not represent a universal TDM. Two versions of the shifting technique (or, the shift transformation) were applied to the preceding model by Hofreiter^74,86^. The DF was based on the fundamental-harmonic (i.e., higher harmonics are neglected) Fourier series expansion of the shifted control (input) signal. A biased relay with hysteresis was used because of practical reasons; however, it was not explicitly included in the algorithm.

As this study concerns a circuit process with heat exchangers, it should also be noted that relay-based identification experiments (followed by a controller design in many cases) have been applied to heat-exchanger systems. For instance, a case study on an autotuning control method for a cross-flow heat exchanger was published in^87^. Jin et al.^88^ presented a Ziegler-Nichols-based method based on using the ultimate gain (instead of on a nonlinear element) to get the sustained oscillations. Some researchers use nonlinear models such as Wiener-type or Hammerstein-type^89^. The reader is also referred to^37^ and references therein.

Let us provide the reader with the motivation to perform the presented research. Models of industrial processes usually include a large number of unknown parameters. Hence, when applying the above-celebrated relay-based identification tests in practice, multiple experiments and/or solutions of nonlinear optimization problems are usually needed (even for linear models). As mentioned above, we focus on a circuit process with heat exchangers with large input–output and internal delays^84^. Considering the simplest single-input single-output case, the derived model includes six non-delay parameters, one internal delay parameter, and two parameters in the input–output relation. Let us call the model as Heat-Exchanger TDM (HETDM) for further presentation. Therefore, any attempt to fit these nine parameter values (e.g., in the frequency domain) requires a good initial guess. Hence, the main idea of the presented research is to perform a two-step relay-feedback identification procedure. The SFOTDM is assumed in the first step, which requires a relatively low computational effort when estimating model parameters’ values. The second step adopts the identified SFOTDM in the sense that dominant characteristic values (poles) of the model coincide with the dominant poles of the analytically-derived HETDM. The pole assignment yields the determination of the characteristic function parameters. These values can either be fixed while remaining model parameters are then set from the single experiment data or they constitute the initial estimation that are further enhanced along with other undetermined parameters that do not depend on poles. As the bridge between the two basic steps, a thorough analysis of pole loci of the SFOTDM resulting in explicit and implicit formulae and a simple graphical procedure is made. This quasi-polynomial root analysis constitutes a substantial contribution to the presented paper.

Relay feedback experiments for both the used models use the standard (i.e., on/off) biased relay for the initial estimation of the ultimate gain and the computation of the process static gain^90^. However, the selected asymmetry is small enough not to affect the applied DF yet sufficient to determine the static gain. The relay with saturation^42^ is applied to enhance the DF evaluation. This nonlinear element can estimate the DF precisely in the ideal case. In the second step (i.e., for the HETDM), single and multiple relay tests are made. The single test is performed to identify the transfer function numerator parameters that are not affected by pole loci. Contrariwise, the multiple test attempts to determine four frequency points via three additional experiments utilizing an artificial delay as per the ATV + technique^38,69,71^. The response ultimate data and the given DF are processed via the well-established Levenberg–Marquardt (LM) method^91^ and the Nelder–Mead (NM) algorithm^92^ to solve a certain nonlinear frequency-based constrained optimization problem. Several numerical scenarios are benchmarked. Namely, LM and NM techniques are compared when using a single relay test to determine transfer function numerator parameters. In addition, the NM algorithm is used to estimate eight model parameters when applying the ATV + test.

Contributions of the presented research can be summarized as follows:

1. Exact analytic rules to determine pole loci of the SFOTDM are derived.
2. Saturated relay feedback experiment is performed on the SFOTDM. The detected pole loci are then set to the initial parameters’ guess for the HETDM (as a sufficiently accurate mathematical model of the circuit system with heat exchangers), which is enhanced via the LM method, under the single relay-test data.
3. Three scenarios are compared to determine the remaining model parameters and further enhance the already estimated ones via the ATV+ technique and the solution of a nonlinear optimization problem using the LM and NM algorithms.
4. An independent determination of numerator and denominator transfer function coefficients along with the pole loci assignment enables to reduce the number of necessary relay test for some of the scenarios.

The rest of the paper is organized as follows. “Methods and techniques” summarizes theoretical fundamentals of (retarded) TDM spectrum and feedback relay-based experiment, the model parameters’ identification using a DF, and the LM and NM methods. “Results” has two fundamental subsections. The first subsection provides the reader with a detailed analysis of the SFOTDM pole loci. The second one presents the HETDM and all steps of determining its parameter values. Namely, the mathematical model of the HETDM is introduced, then the reader is acquainted with the poles assignment, the transfer function numerator estimation using a single relay test, and the complete model parameters estimation via the ATV + technique. In “Discussion”, the obtained results are discussed. Finally, “Conclusions” concludes the paper.

The standard notation is used throughout the paper, i.e., ${\mathbb{C}},{\mathbb{N}},{\mathbb{R}}$ denote the sets of complex, natural (excluding zero) and real numbers, respectively, ${\mathbb{R}}_{ + }^{n}$ expresses the *n*-dimensional Euclidean space of positive real-valued vectors, ${\text{Re}} \left( s \right)$ and ${\text{Im}} \left( s \right)$ mean the real and imaginary parts of some $s \in {\mathbb{C}}$, respectively. Superscript $T$ denotes the vector (matrix) transpose.

## Methods and techniques

### Retarded quasi-polynomial and its spectrum

Let us concisely introduce the Retarded Quasi-Polynomial (RQP) form and its spectrum, i.e., the zero points^9,14,15^. A RQP has the following form

1 $$q\left( s \right) = s^{n} + \sum\nolimits_{i = 0}^{n - 1} {\sum\nolimits_{j = 0}^{{m_{i} }} {q_{ij} s^{i} {\text{e}}^{{ - \tau_{ij} s}} } }$$

where $s \in {\mathbb{C}}$ is the Laplace transform variable, $q_{ij} \in {\mathbb{R}}$ are non-delay parameters, $\tau_{ij} \in {\mathbb{R}}_{ + }$ with $\tau_{i0} = 0$ represent delays, and $n \in {\mathbb{N}}$ means the RQP order (of derivative).

#### Definition 1

The *RQP spectrum* is the set of RQP zeros, i.e.,

2 $$\Sigma : = \left\{ {s:q\left( s \right) = 0} \right\}$$

#### Proposition 1

It holds for $\Sigma$ that

1. If exist $i \ge 0,j > 0$ such that $q_{ij} ,\tau_{ij} \ne 0$, then $\left| \Sigma \right| = \infty$ (i.e., the RQP spectrum is infinite).
2. RQP zeros $s_{k} \in \Sigma$ are isolated and function ${\mathbb{R}}^{{\sum\nolimits_{i = 0}^{n - 1} {m_{i} } }} { \mathrel\backepsilon }\left( {q_{ij} ,\tau_{ij} } \right) \mapsto s_{k} \in {\mathbb{C}}$ is continuous.
3. For any finite $\gamma \in {\mathbb{R}}$, the subset $\Sigma_{R} = \left\{ {s \in \Sigma :{\text{Re}} s > \gamma } \right\}$ is finite, while $\Sigma_{L} = \left\{ {s \in \Sigma :{\text{Re}} s \le \gamma } \right\}$ is infinite. □

Note that the relation $\left( {q_{ij} ,\tau_{ij} } \right) \mapsto s_{k}$ is not necessarily smooth; namely, in points where a multiple real root bifurcates into a complex pair.

#### Definition 2

The RQP spectral abscissa is defined as

3 $$\alpha_{\Sigma } : = \sup \left\{ {{\text{Re}} s:s \in \Sigma } \right\}$$

### Relay-based parameter identification

**Figure 1:**
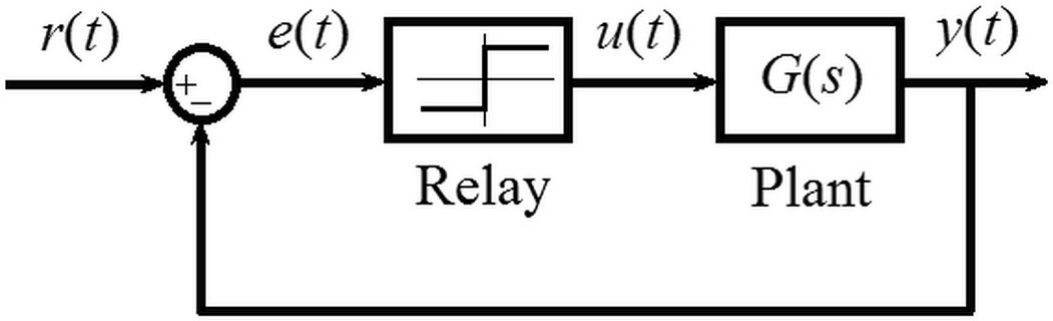
A framework scheme of the relay feedback identification experiment.

As introduced above, experimental plant identification using the relay (or another simple nonlinear element) method represents a widely used technique in various engineering and industrial applications. Consider a plant (the model of which is to be identified) under a relay feedback control, as depicted in Fig. 1. In the figure, $r\left( t \right),e\left( t \right),u\left( t \right)$, and $y\left( t \right)$ mean the reference, control error, manipulated input and controlled output variables, and $G\left( s \right)$ stands for the actual plant (process) dynamics. The choice of $r\left( t \right)$ (usually of a constant value) enables to set the operating point.

If the relay parameters are appropriately set, the closed-loop system reaches sustained oscillations of period $T_{osc}$ in a finite time. If the relay element does not cause a phase lag, the corresponding angular frequency $\omega_{osc} = 2\pi /T_{osc}$ is supposed to be close to the so-called ultimate frequency $\omega_{u}$ for which ${\text{Im}} G\left( {{\text{j}}\omega_{u} } \right) = 0$ (more precisely $\measuredangle G\left( {{\text{j}}\omega_{u} } \right) = - \pi$), ${\text{j}}^{2} = - 1$. However, as a model $G_{m} \left( s \right)$ cannot express the true dynamics $G\left( s \right)$ exactly, it generally holds that $\omega_{osc} \ne \omega_{u}$. Whenever the relay exposes a phase lag, $\omega_{osc} < \omega_{u}$.

By adopting the idea of the DF, one point $G\left( {{\text{j}}\omega_{osc} } \right) \in {\mathbb{C}}$ can be estimated, i.e., two parameters of $G_{m} \left( s \right)$ can be determined. The relay DF, $N\left( \cdot \right) \in {\mathbb{C}}$, can be considered as a linear approximation of the relay gain. It is usually derived using a consideration that $e\left( t \right)$ is a harmonic signal and $u\left( t \right)$ is subject to a truncated Fourier series expansion. Then, for the sustained oscillations, it holds that

4 $$N\left( \cdot \right)G_{m} \left( {{\text{j}}\omega_{osc} } \right) = - 1 + 0{\text{j}} \Leftrightarrow \left\{ {\begin{array}{*{20}c} {\left| {N\left( \cdot \right)G_{m} \left( {{\text{j}}\omega_{osc} } \right)} \right| = 1} \\ {\measuredangle \left( {N\left( \cdot \right)G_{m} \left( {{\text{j}}\omega_{osc} } \right)} \right) = - \pi } \\ \end{array} } \right.$$

which enables to estimate parameters of $G_{m} \left( s \right)$. Note that (4) can be graphically interpreted as the intersection of the Nyquist plot of $G_{m} \left( s \right)$ with the horizontal line $- N^{ - 1} \left( \cdot \right)$. The DF depends on the amplitude $A$ of $e\left( t \right)$ oscillations and some other relay setting parameters.

### On/off relay test

**Figure 2:**
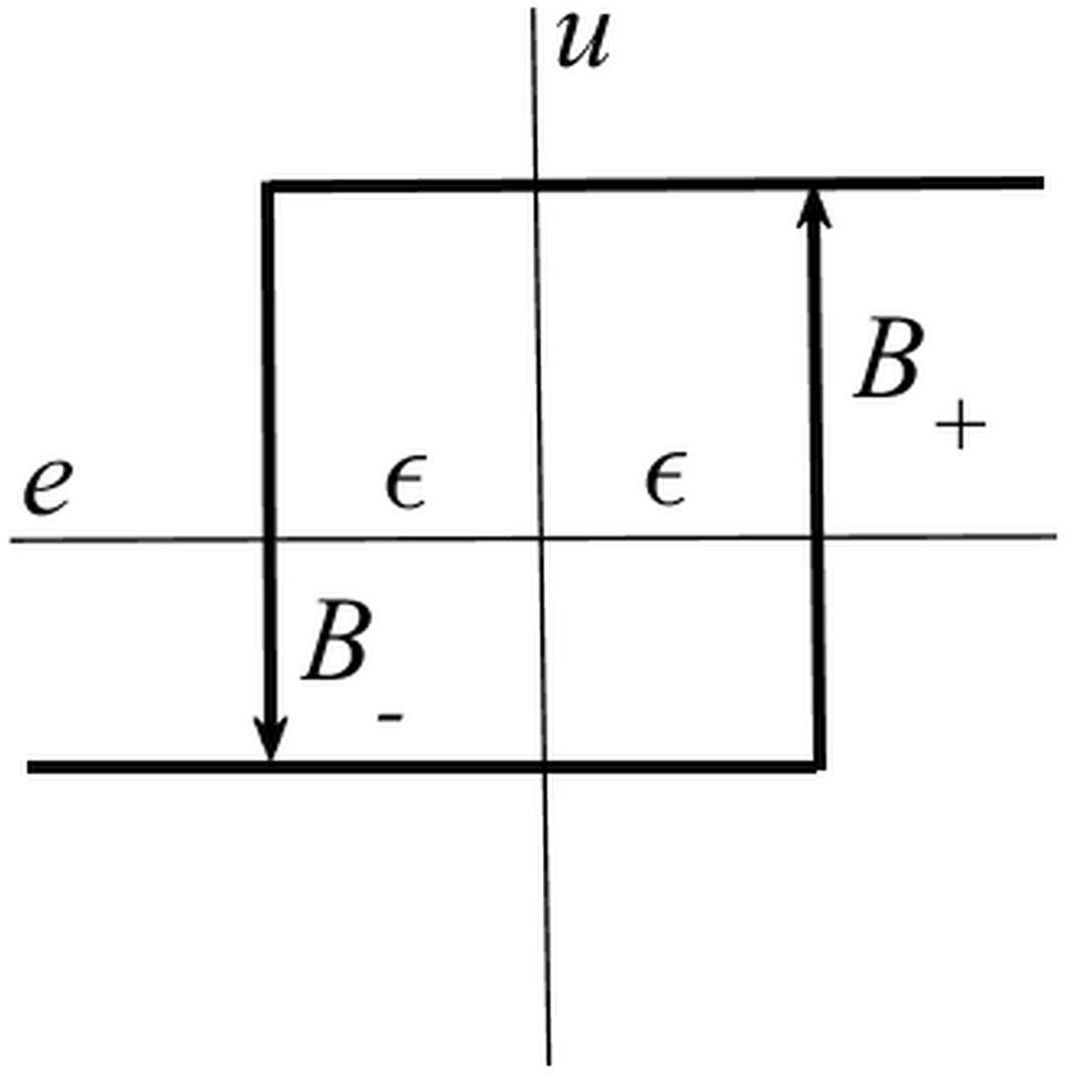
The static characteristics of the asymmetrical biased relay.

**Figure 3:**
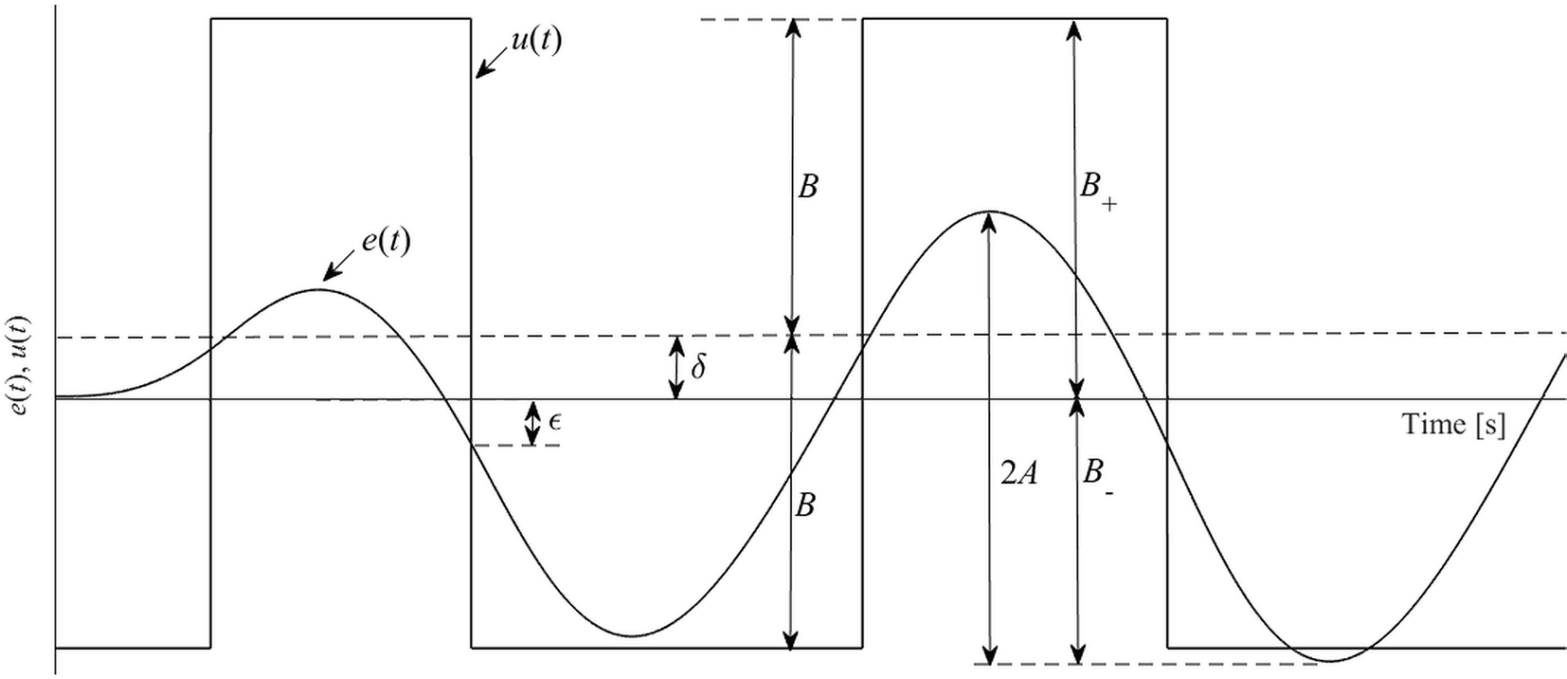
Sustained oscillations using the asymmetrical biased relay.

Let us consider an asymmetrical biased two-level relay. Its static characteristics and the corresponding sustained oscillations (limit cycles) are displayed in Figs. 2 and 3, respectively.

In the figures, $B_{ + } ,B_{ - }$ are upper and lower relay output levels, respectively, for which the bias (shift) parameter reads $\delta = \left| {B_{ + } - B_{ - } } \right|/2$, and $\varepsilon \ge 0$ expresses the hysteresis parameter. The particular DF is

5 $$\begin{gathered} N\left( {A,\delta ,\varepsilon } \right) = \frac{4B}{{\pi A}}\sqrt {1 - \left( {\frac{\delta }{A}} \right)^{2} } \left( {\sqrt {1 - \left( {\frac{\varepsilon }{A}} \right)^{2} } - {\text{j}}\frac{\varepsilon }{A}} \right), \hfill \\ \delta ,\varepsilon < A \hfill \\ \end{gathered}$$

where $B = \left( {B_{ + } + B_{ - } } \right)/2$, see, e.g.^67,68,73^. Then, the ideal on/off relay gives rise to $N\left( {A,0,0} \right) = N\left( A \right) = 4B/\left( {\pi A} \right)$.

In practice, the setting $\varepsilon \ne 0$ is suitable when the feedback signal is affected by noise so that the switching relay rate can be reduced. The advantage of the option $\delta \ne 0$ lies, i.a., in the possibility to estimate the process static gain $k = G_{m} \left( 0 \right) = G\left( 0 \right)$ as

6 $$k = \frac{{\int_{{t_{0} }}^{{t_{0} + T_{{osc}} }} {y\left( \theta \right)} d\theta }}{{\int_{{t_{0} }}^{{t_{0} + T_{{osc}} }} {u\left( \theta \right)} d\theta }}$$

for $t_{0}$ satisfying that sustained oscillations start for some $t < t_{0}$^35^.

Purposefully induced asymmetry can also be used to estimate and attenuate the load disturbance^42^. However, it may stop oscillations so that the relay test fails. In addition, model parameter identification with asymmetric relay yields an estimation error of up to 40% in a FOPDT case^50^.

### Relay with saturation

Estimating the critical point at $\omega_{u}$ (or any other $\omega_{osc}$) does not provide an accurate enough parameter estimation for some processes, e.g., for those with significant time delays. For instance, an error of 23% for FOPDT models was reported^59^.

**Figure 4:**
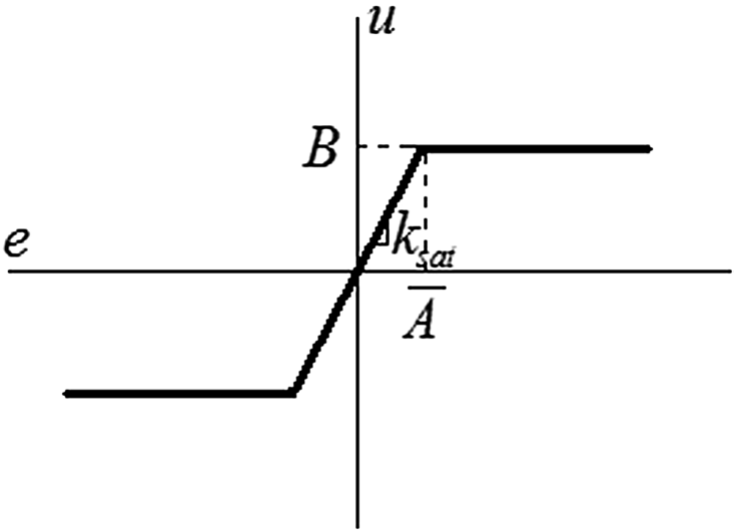
The static characteristics of the saturation relay.

**Figure 5:**
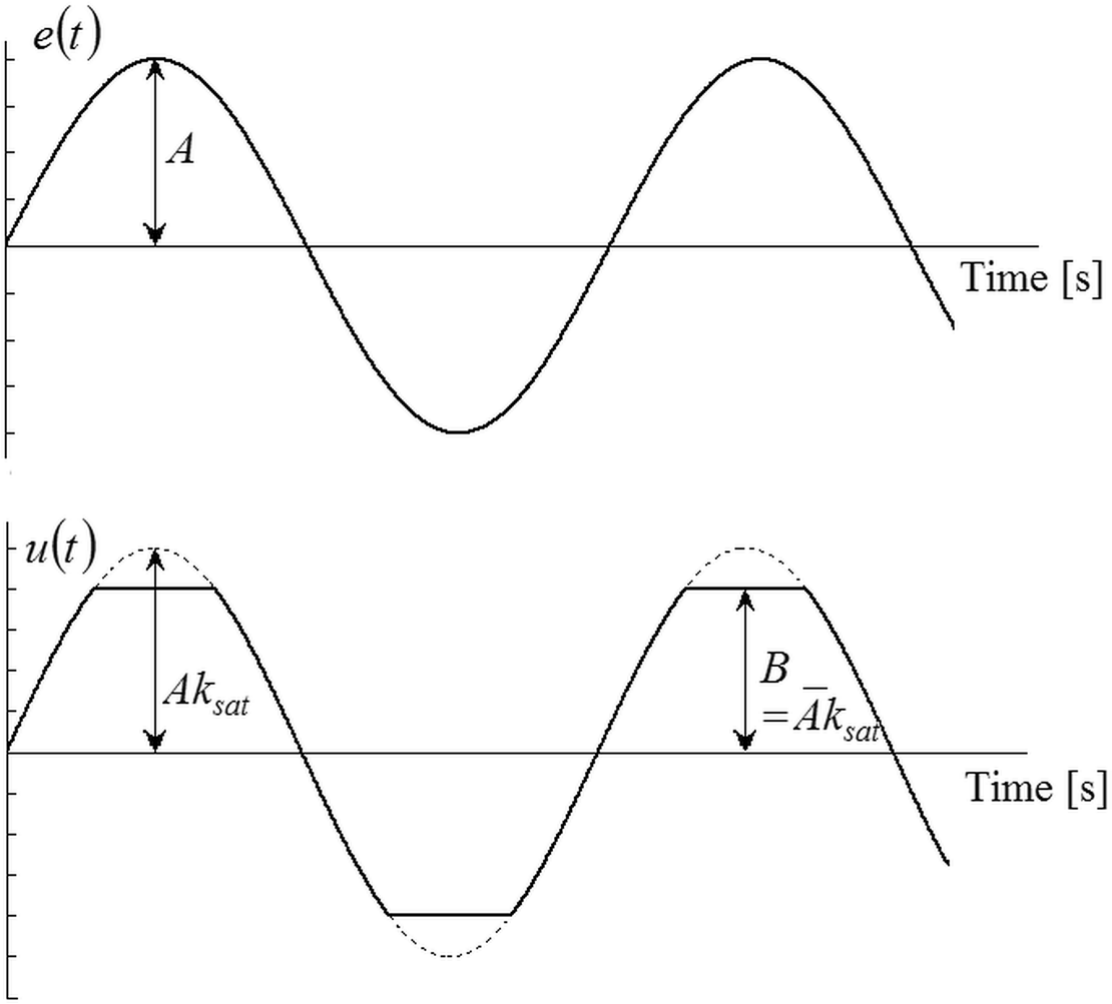
Sustained oscillations using the saturation relay.

Model parameters identification can be improved by using saturation relay^35,59^. Its static characteristics and a sketch of the corresponding sustained oscillations (under the assumption of a harmonic output variable) are depicted in Figs. 4 and 5.

The saturation relay does not cause an abrupt step change at $e\left( t \right) = \pm \varepsilon$, yet it provides a smooth transient around zero. The relay input $e\left( t \right)$ is multiplied by $k_{sat}$ resulting in the relay output $u\left( t \right)$ up to the limit $B = k_{sat} \overline{A}$*.* The corresponding DF reads

7 $$N_{sat} \left( {A,\overline{A}} \right) = \frac{2B}{\pi }\left( {\frac{1}{{\overline{A}}}\sin^{ - 1} \left( {\frac{{\overline{A}}}{A}} \right) + \frac{{\sqrt {A^{2} - \overline{A}^{2} } }}{{A^{2} }}} \right)$$

Ideally, if the gain $k_{sat}$ is set optimally (i.e., $A = \overline{A}$), input and output signals has the same shape; hence, the DF $N_{sat} \left( {A,\overline{A}} \right) = k_{sat}$ is exact. However, in real conditions, $u\left( t \right)$ has a shape of the truncated sinusoidal wave with upper and lower limits. Not that the limit case $k_{sat} \to \infty$ yields the ideal relay, i.e. $N_{sat} \left( {A,0} \right) = N\left( {A,0,0} \right)$.

A saturation relay test should follow the standard relay experiment (see the preceding subsection). Once $k_{osc} = N\left( {A, \cdot } \right)$ is found, then it is set $k_{sat} = k_{\min } k_{osc} ,k_{\min } > 1$. Originally, it was suggested to take $k_{\min } = 1.4$^59^. The higher the value is, the closer to the two-level signal $u\left( t \right)$ is. Contrariwise, smaller values of $k_{\min }$ force $u\left( t \right)$ to be closer to sinus-like waves; however, the relay takes a longer time or even can fail to generate sustained oscillation.

### ATV + technique

One of the fundamental drawbacks of the original relay feedback test is that only a single point of the frequency characteristics can be determined, which allows estimating only two model parameters. The ATV + technique^38,69,71^ introduces an artificial delay $\tau_{a} > 0$ to the serial link between the relay and the process.

Every single value of $\tau_{a}$ causes the phase lag of $\varphi_{a} = \overline{\omega }_{osc} \tau_{a}$ where $\overline{\omega }_{osc}$ means the corresponding angular frequency of sustained oscillations when the delay applies here. Then, the overall phase shift attributed to the process reads

8 $$\measuredangle G\left( {{\text{j}}\overline{\omega }_{osc} } \right) = - \pi - \measuredangle N\left( \cdot \right) + \varphi_{a}$$

Hence, by detecting $\overline{\omega }_{osc}$ and the corresponding amplitude $\overline{A}$, another point on the process (model) Nyquist curve can be determined. Obviously, whenever a number of $N$ model parameters are needed to be resolved, then $\left\lceil {N/2 - 1} \right\rceil$ distinct $\tau_{a}$ s is required, where $\left\lceil \cdot \right\rceil$ means the ceiling function (i.e., the rounding upward to the nearest integer).

The original setting^69^ comes from the following idea. The goal is to identify a point located at 45° distance from the negative real axis, i.e., $\varphi_{a} = \pi /4$ (under the assumption that $\measuredangle N\left( \cdot \right) = 0$). This point is expected to occur at frequency $\overline{\omega }_{osc} = 3/5\omega_{u}$. It eventually yields the following condition and the setting result

9 $$\tau_{a} \frac{3}{5}\omega_{u} = \frac{\pi }{4} \Rightarrow \tau_{a} = \frac{5\pi }{{12}}\omega_{u}$$

The disadvantage of this technique is the prolongation of the relay feedback experiment. However, if the initial conditions are sustained oscillations, it lasts a significantly shorter time to restore the oscillations than starting from a constant steady state.

### Parameter optimization methods

To solve (2) for given roots $s_{k}$, (4), and (8), two well-established optimization algorithms are adopted. Their concise description to acquaint the reader follows.

### Levenberg–Marquardt method

Consider a set of nonlinear differentiable functions ${\mathbf{f}} = \left( {f_{1} ,f_{2} , \ldots f_{n} } \right)^{T}$, ${\mathbb{C}} \times {\mathbb{R}}^{m} \mathrel\backepsilon \left( {x,{\mathbf{p}}} \right) \mapsto f_{i} \left( {x,{\mathbf{p}}} \right) \in {\mathbb{R}}$, $i = 1,2,\ldots ,n$, where $x$ is a function variable, and ${\mathbf{p}} = \left( {p_{1} ,p_{2} ,\ldots ,p_{m} } \right)^{T} \in {\mathbb{R}}^{m}$ expresses the set of function parameters. Then, the set of algebraic functions

10 $${\mathbf{f}} = {\mathbf{0}}$$

where ${\mathbf{0}} = \left( {0,0, \ldots ,0} \right)^{T}$, can be iteratively solved via

11 $$\begin{gathered} {}^{k + 1}{\mathbf{p}} = {}^{k}{\mathbf{p}} + \Delta {}^{k}{\mathbf{p}} \hfill \\ \Delta {}^{k}{\mathbf{p}} = - \left( {{}^{k}{{\varvec{\Xi}}} + {}^{k}\lambda {\text{diag}}\left( {{}^{k}{{\varvec{\Xi}}}} \right)} \right)^{ - 1} \left[ {{\mathbf{J}}\left( {x,{}^{k}{\mathbf{p}}} \right)} \right]^{T} {\mathbf{f}}\left( {x,{}^{k}{\mathbf{p}}} \right) \hfill \\ \end{gathered}$$

where

12 $${}^{k}{{\varvec{\Xi}}} = \left[ {{\mathbf{J}}\left( {{\mathbf{f}}\left( {x,{}^{k}{\mathbf{p}}} \right)} \right)} \right]^{T} {\mathbf{J}}\left( {{\mathbf{f}}\left( {x,{}^{k}{\mathbf{p}}} \right)} \right)$$

${\mathbf{J}}$ means the Jacobian of ${\mathbf{f}}$ with respect to ${\mathbf{p}}$, $k$ expresses the iteration step, and $\lambda > 0$ is an adjustable parameter (the so-called damping factor)^93^. Solution (11) and (12) attempts to solve the nonlinear least-squares problem, i.e.,

13 $${\mathbf{p}}_{opt} = \lim_{k \to \infty } {}^{k}{\mathbf{p}} = \mathop {\arg }\limits_{{\mathbf{p}}} \min \sum\nolimits_{i = 1}^{n} {\left( {f_{i} \left( {x,{\mathbf{p}}} \right)} \right)^{2} } = :\mathop {\arg }\limits_{{\mathbf{p}}} {\text{Res}}\left( {\mathbf{p}} \right)$$

The value of $\lambda$ (the so-called damping factor) may vary during iterations. One of the framework strategies is to decrease its value as the residual sum on the right-hand side of (13) (${\text{Res}}\left( {\mathbf{p}} \right)$) decreases, and vice versa. Let us introduce the multiplicative factor $\kappa > 0$ as ${}^{k + 1}\lambda = {}^{k}\lambda \kappa$. Particular choices of ${}^{1}\lambda ,{}^{1}{\mathbf{p}}$, and $\kappa$ are discussed in “Relay-based parameter identification of heat-exchanger process”.

A disadvantage of the LM algorithm is that the solution may converge to a local minimum (as for other Newton-type methods) or it may even diverge (especially, if $\lambda$ is set inappropriately).

### Nelder–Mead method

Assume an unconstrained optimization problem, first

14 $$\min \,f\left( {\mathbf{p}} \right) \in {\mathbb{R}}{,}\,{\mathbf{p}} \in {\mathbb{R}}^{m}$$

The idea of the NM method^92^ is to iteratively search for the optimal solution by moving a variable-shape simplex in the space of ${\mathbf{p}}$. The simplex vertices represent the so-called test points. Once the initial simplex ${}^{1}S = \left( {{}^{1}{\mathbf{p}}_{1} ,{}^{1}{\mathbf{p}}_{2} ,\ldots ,{}^{1}{\mathbf{p}}_{m + 1} } \right)$ is selected, its vertices are re-ordered such that $f\left( {{}^{1}{\mathbf{p}}_{i} } \right) \le f\left( {{}^{1}{\mathbf{p}}_{i + 1} } \right),i = 1,2,\ldots m$ (i.e., ${}^{1}{\mathbf{p}}_{1}$ represents the best solution estimation) and set $k = 1$. Then, the center of the subvector ${}^{k}\tilde{S} = \left( {{}^{k}{\mathbf{p}}_{1} ,{}^{k}{\mathbf{p}}_{2} ,\ldots ,{}^{k}{\mathbf{p}}_{m} } \right)$ is computed

15 $${}^{k}{\mathbf{p}}_{c} = \frac{1}{m}\sum\nolimits_{i = 1}^{m} {{}^{k}{\mathbf{p}}_{i} }$$

The worst-valued vertex is reflected through ${}^{k}{\mathbf{p}}_{c}$ as ${}^{k}{\mathbf{p}}_{r} = {}^{k}{\mathbf{p}}_{c} + \gamma_{r} \left( {{}^{k}{\mathbf{p}}_{c} - {}^{k}{\mathbf{p}}_{n + 1} } \right),\,\,\gamma_{r} > 0$. Then, four scenarios can happen:

1. If $f\left( {{}^{k}{\mathbf{p}}_{1} } \right) \le f\left( {{}^{k}{\mathbf{p}}_{r} } \right) < f\left( {{}^{k}{\mathbf{p}}_{m} } \right)$, then set a new simplex ${}^{k + 1}S = \left( {{}^{k}{\mathbf{p}}_{1} ,{}^{k}{\mathbf{p}}_{2} ,\ldots ,{}^{k}{\mathbf{p}}_{m} ,{}^{k}{\mathbf{p}}_{r} } \right)$.
2. If $f\left( {{}^{k}{\mathbf{p}}_{r} } \right) < f\left( {{}^{k}{\mathbf{p}}_{1} } \right)$, compute the expanded point ${}^{k}{\mathbf{p}}_{e} = {}^{k}{\mathbf{p}}_{c} + \gamma_{e} \left( {{}^{k}{\mathbf{p}}_{r} - {}^{k}{\mathbf{p}}_{c} } \right),\,\,\gamma_{e} > 1$. On condition that $f\left( {{}^{k}{\mathbf{p}}_{e} } \right) < f\left( {{}^{k}{\mathbf{p}}_{1} } \right)$, set ${}^{k + 1}S = \left( {{}^{k}{\mathbf{p}}_{1} ,{}^{k}{\mathbf{p}}_{2} ,\ldots ,{}^{k}{\mathbf{p}}_{m} ,{}^{k}{\mathbf{p}}_{e} } \right)$, else ${}^{k + 1}S = \left( {{}^{k}{\mathbf{p}}_{1} ,{}^{k}{\mathbf{p}}_{2} ,\ldots ,{}^{k}{\mathbf{p}}_{m} ,{}^{k}{\mathbf{p}}_{r} } \right)$.
3. If $f\left( {{}^{k}{\mathbf{p}}_{m} } \right) \le f\left( {{}^{k}{\mathbf{p}}_{r} } \right) < f\left( {{}^{k}{\mathbf{p}}_{m + 1} } \right)$, the outer contraction is done as ${}^{k}{\mathbf{p}}_{oc} = {}^{k}{\mathbf{p}}_{c} + \gamma_{oc} \left( {{}^{k}{\mathbf{p}}_{r} - {}^{k}{\mathbf{p}}_{c} } \right)$, $0 < \gamma_{oc} < 1$. Whenever $f\left( {{}^{k}{\mathbf{p}}_{oc} } \right) < f\left( {{}^{k}{\mathbf{p}}_{r} } \right)$, set ${}^{k + 1}S = \left( {{}^{k}{\mathbf{p}}_{1} ,{}^{k}{\mathbf{p}}_{2} ,\ldots ,{}^{k}{\mathbf{p}}_{m} ,{}^{k}{\mathbf{p}}_{oc} } \right)$, else perform the shrinkage as

16 $${}^{k + 1}S = \gamma_{s} {}^{k}S,\,\,0 < \gamma_{s} < 1$$

4. If $f\left( {{}^{k}{\mathbf{p}}_{r} } \right) \ge f\left( {{}^{k}{\mathbf{p}}_{m + 1} } \right)$, compute the inner contraction ${}^{k}{\mathbf{p}}_{ic} = {}^{k}{\mathbf{p}}_{c} + \gamma_{ic} \left( {{}^{k}{\mathbf{p}}_{m + 1} - {}^{k}{\mathbf{p}}_{c} } \right)$, $0 < \gamma_{ic} < 1$. On condition that $f\left( {{}^{k}{\mathbf{p}}_{ic} } \right) < f\left( {{}^{k}{\mathbf{p}}_{m + 1} } \right)$, set ${}^{k + 1}S = \left( {{}^{k}{\mathbf{p}}_{1} ,{}^{k}{\mathbf{p}}_{2} ,\ldots ,{}^{k}{\mathbf{p}}_{m} ,{}^{k}{\mathbf{p}}_{ic} } \right)$, else shrink the simplex using (16).

Then $k = k + 1$, re-order simplex vertices, and calculate (15), etc.

If, however, inequality constraints

17 $$g_{j} \left( {{\tilde{\mathbf{p}}}} \right) < 0,\,j = 1,2,\ldots ,n$$

on a subset ${\tilde{\mathbf{p}}} \subseteq {\mathbf{p}}$ are required, one may use the concept of barrier functions. That is, instead of the objective function $f\left( {\mathbf{p}} \right)$ as in (14), the extended function $\Phi \left( {\mathbf{p}} \right)$ is subject to the optimization procedure

18 $$\Phi \left( {\mathbf{p}} \right) = f\left( {\mathbf{p}} \right) + \beta f_{b} \left( {{\tilde{\mathbf{p}}}} \right)\,$$

where $\beta > 0$ and $f_{b} \left( {{\tilde{\mathbf{p}}}} \right) > 0$ must be sufficiently small as soon as all $g_{j} \left( {{\tilde{\mathbf{p}}}} \right) \ll 0$; otherwise, the value of $f_{b} \left( {{\tilde{\mathbf{p}}}} \right)$ increases considerably until $f_{b} \left( {{\tilde{\mathbf{p}}}} \right) \to \infty$ as $g_{j} \left( {{\tilde{\mathbf{p}}}} \right) \to 0^{ - }$.

## Results

### Root loci analysis of the simple quasi-polynomial

In this subsection, a thorough zero loci analysis of the SFOTDM^12^ is provided. The derived results then serve for the pole assignment of the HETDM giving rise to the initial parameters setting of its CE (see “Parameter estimation of the heat-exchanger process model via pole assignment”). The model reads

19 $$G_{SFOTDM} \left( s \right) = \frac{k}{{Ts + {\text{e}}^{ - \vartheta s} }}{\text{e}}^{ - \tau s}$$

where $$0 < T,\vartheta ,\tau ,k < \infty$$.

Although pole loci properties of the SFOTDM were studied in the past, according to the authors’ best knowledge, a complete image and a thorough exact guide on finding the dominant subset of its spectrum has not been provided yet. For instance, Marshal, Gorecki, Walton, and Korytowski^94^ studied a generalized characteristic RQP of the SFOTDM with relative parameters ($T = 1,\Theta = \vartheta /T,\theta = \tau /T$), and they determined ranges in which the model is asymptotically stable, aperiodic, and periodic. Moreover, intersections of pole loci trajectories with the imaginary axis for the generalized model were determined. Analogous conditions for which the model is stable, overdamped, critically damped, and underdamped were presented in^95^. Asymptotic behavior of pole loci trajectories in infinity and nearby the imaginary axis were also studied in^96^.

Hence, our aim is to analyze the solution (or its rightmost subset) of the CE

20 $${\text{CE}}_{SFOTDM} :q_{SFOTDM} \left( s \right) = Ts + {\text{e}}^{ - \vartheta s} = 0$$

in ${\mathbb{C}}$ and provide the reader with a simple guide how to compute these pole loci.

#### Lemma 1

^12,94,96^ All solutions of (20) lie in the open left half complex plane (LHP) if and only if

21 $$\frac{1}{T} \in \left( {0,\frac{\pi }{2\vartheta }} \right)$$

□

Result (21) can also be formulated as $\Theta \in \left( {0,0.5\pi } \right)$.

#### Lemma 2

^12,94,96^. There exists a double real root $s_{1,2} = - 1/\vartheta$ in the spectrum of $q_{SFOTDM} \left( s \right)$ if and only if

22 $$\frac{1}{T} = \frac{1}{{{\text{e}} \vartheta }}$$

In addition, there does not exist a solution of (20) or its pair $s_{i} = \alpha + \omega {\text{j,}}\,\,\overline{s}_{i} = \alpha - \omega {\text{j}}$ with

23 $$\alpha \in \left( { - \frac{1}{\vartheta },0} \right),\omega \ge 0$$

where the bar denotes the complex conjugate. □

Result (22) can also be formulate as $\Theta = {\text{e}}^{ - 1}$. Let us introduce relative real and imaginary parts of a quasi-polynomial root as $\overline{\alpha } = - \vartheta \alpha$, $\overline{\omega } = \vartheta \omega$, respectively. Then the range (23) becomes

24 $$\overline{\alpha } \in \left( {0,1} \right),\overline{\omega } \ge 0$$

Lemma 2 means that $q_{SFOTDM} \left( s \right)$ has the rightmost double real root at $\overline{\alpha } = 1$ for $\Theta = {\text{e}}^{ - 1}$.

#### Lemma 3

^96^. The double dominant (i.e., rightmost) real root $s_{1,2} = - 1/\vartheta$ (i.e., $\overline{\alpha } = 1$) becomes a complex conjugate pair for $\Theta_{{\lim \delta \to 0^{ + } }} = {\text{e}}^{ - 1} + \delta$. Contrariwise, the double real root becomes a pair of single real roots for $\Theta_{{\lim \delta \to 0^{ + } }} = {\text{e}}^{ - 1} - \delta$. □

#### Theorem 1

$q_{SFOTDM} \left( s \right)$ has a real dominant zero in the LHP for $\Theta = \left( {0,{\text{e}}^{ - 1} } \right)$ and a complex conjugate rightmost pair in the LHP for $\Theta = \left( {{\text{e}}^{ - 1} ,0.5\pi } \right)$, where the particular root abscissa is within the range (23) (or (24), equivalently). □

#### Proof

 From Lemma 1, a negative root abscissa exists only for (23). If $\Theta$ ranges from 0 to ${\text{e}}^{ - 1}$, the rightmost real root moves from $\overline{\alpha } = 0$ to $\overline{\alpha } = 1$ due to Lemma 2 and Lemma 3. From Lemma 2, it is also known that there is the rightmost double real root for $\Theta = {\text{e}}^{ - 1}$ that bifurcates into a conjugate pair for $\Theta = \left( {{\text{e}}^{ - 1} ,0.5\pi } \right)$. Eventually, this pair reaches the imaginary axis again for $\Theta = 0.5\pi$ as the only (i.e., the rightmost) quasi-polynomial root pair due to Lemma 1. ■

In the following part of the subsection, dominant (and other) SFOTDM pole loci are investigated.

#### Lemma 4

^96^. Whenever $s_{1,2} = \pm \omega {\text{j,}}\,\omega \ne 0$, it holds that $\omega = \frac{1}{T} = \frac{{\left( {2k + 1} \right)\pi }}{2\vartheta }$ (i.e., $\overline{\omega } = \Theta = \frac{2k + 1}{2}\pi$),$k = 0,1,2,\ldots$. □

#### Lemma 5

The double real $s_{1,2} = - 1/\vartheta$ (i.e., $\overline{\alpha } = 1$) in $\Theta = {\text{e}}^{ - 1}$ is the only multiple real root of $q_{SFOTDM} \left( s \right)$. □

#### Proof

 The double real root $s_{1,2} = \alpha$ must satisfy

25 $$\left. {q_{SFOTDM} \left( s \right)} \right|_{s = \alpha } = \left. {q^{\prime}_{SFOTDM} \left( s \right)} \right|_{s = \alpha } = 0$$

where $q^{\prime}_{SFOTDM} \left( s \right) = \frac{{{\text{d}}q_{SFOTDM} \left( s \right)}}{{{\text{d}}s}}$. Conditions (25) read

26 $$\begin{aligned} & T\alpha + {\text{e}}^{ - \vartheta \alpha } = 0 \\ & T - \vartheta {\text{e}}^{ - \vartheta \alpha } = 0 \\ \end{aligned}$$

The first formula of (26) can be rewritten as

27 $$\begin{aligned} & \alpha + \frac{1}{T}{\text{e}}^{ - \vartheta \alpha } = 0 \Leftrightarrow - \frac{{\overline{\alpha }}}{\vartheta } + \frac{1}{T}{\text{e}}^{ - \vartheta \alpha } = 0 \Leftrightarrow - \overline{\alpha } + \Theta {\text{e}}^{ - \vartheta \alpha } = 0 \\ & \Rightarrow \Theta = \frac{{\overline{\alpha }}}{{{\text{e}}^{{\overline{\alpha }}} }} \\ \end{aligned}$$

The latter condition in (26) agrees with

28 $$1 - \Theta {\text{e}}^{{\overline{\alpha }}} = 0 \Leftrightarrow \Theta = \frac{1}{{{\text{e}}^{{\overline{\alpha }}} }}$$

By comparison (27) and (28), it can be deduced that $\overline{\alpha } = 1$ is the only double real root.

Now we must show that it is the only multiple root for any finite $\Theta$. Such a root must satisfy (25) and simultaneously

29 $$\left. {q^{\left( k \right)}_{{_{SFOTDM} }} \left( s \right)} \right|_{s = \alpha } = \left( { - 1} \right)^{k} \vartheta^{k - 1} \Theta {\text{e}}^{{\overline{\alpha }}} = 0,\,\,k = 2,3,\ldots$$

Since $\vartheta \ne 0$, only two solutions of (29) exist

30 $$\Theta = 0\,\,\,\,{\text{or}}\,\,\,\,\overline{\alpha } \to - \infty$$

The former solution in (30) yields the stability border due to Lemma 1 and $\overline{\alpha } \to \infty$ from (27) and (28), which indicates a root at infinity. It is, however, in contradiction with (29). The latter possibility in (30) gives $\Theta \to \infty$ from (27) but $\Theta \to - \infty$ from (28), which yields a contradiction again.

As an alternative of the proof, one can easily deduce that (28) and (29) are in contradiction.■

#### Lemma 6

 Equation (20) can have only two real solutions (counting multiplicity). □

#### Proof

Lemma 6 implies from Lemma 5 directly due to the root continuity (see Proposition 1). That is, a complex conjugate zeros of $q_{SFOTDM} \left( s \right)$ can bifurcate in a pair of distinct real roots only through a multiple pair.

**Figure 6:**
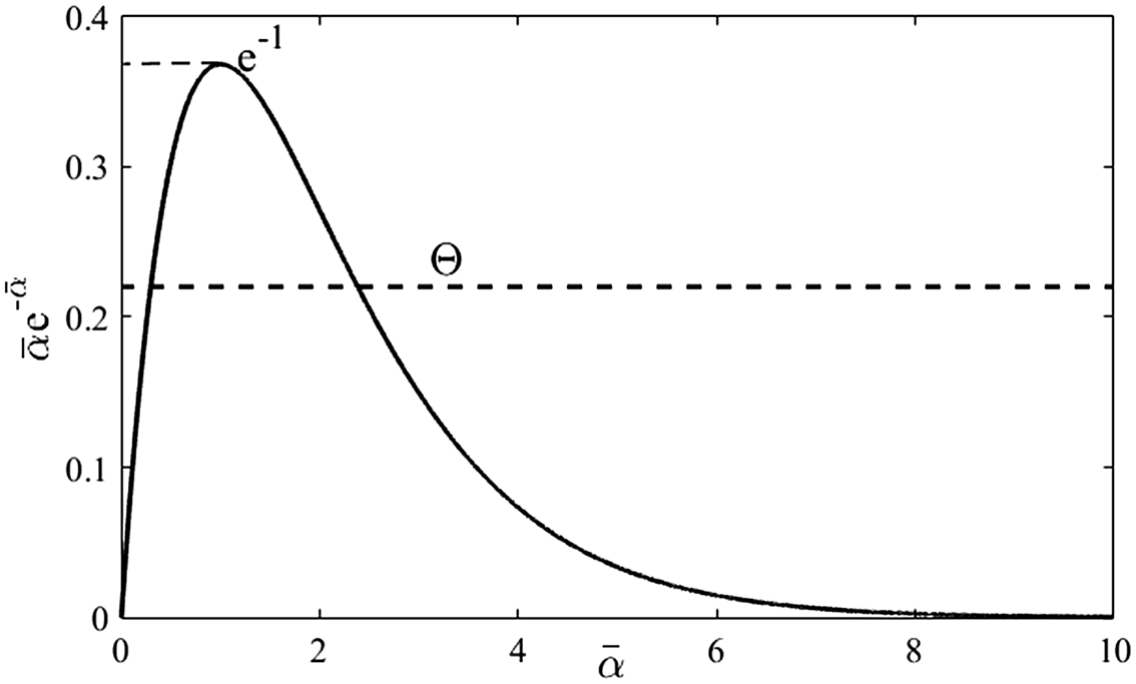
Intersections of a constant function $\Theta$ and $\overline{\alpha }{\text{e}}^{{ - \overline{\alpha }}}$ (Lemma 6).

Alternatively, distinct real solutions of (20) satisfy (27). Function $\overline{\alpha } \mapsto \overline{\alpha }{\text{e}}^{{ - \overline{\alpha }}}$ is unimodal with local and global maximum in $\overline{\alpha } = 1$ and the function value $1/{\text{e}}$. This point agrees with Lemma 5. Otherwise, the function has two distinct intersections with the constant function $\Theta \in \left( {0,{\text{e}}^{ - 1} } \right)$ for $\overline{\alpha } \in \left( {0,\infty } \right]$. Hence, there is no real solution of (20) for $\Theta > {\text{e}}^{ - 1}$. The situation is illustrated in Fig. 6.■

#### Theorem 2

Let $\Theta$ has a positive finite value. Then,

- If $\Theta > {\text{e}}^{ - 1}$, complex conjugate (single) zeros $s_{i} = \alpha + \omega {\text{j,}}\,\overline{s}_{i} = \alpha - \omega {\text{j}}$,$\omega > 0$, of $q_{SFOTDM} \left( s \right)$ are given by all solutions of the set of equations
  31 $$\overline{\alpha } = \frac{{\overline{\omega }}}{{\tan \overline{\omega }}},\,\overline{\omega } \ne k\pi ,\,k = 1,2,\ldots$$
  32 $$\overline{\alpha } = \ln \left( {\frac{{\overline{\omega }}}{{\Theta \sin \overline{\omega }}}} \right),\overline{\alpha } \in {\mathbb{R}}$$
- If $\Theta \in \left( {0,{\text{e}}^{ - 1} } \right)$, complex conjugate zeros of $q_{SFOTDM} \left( s \right)$ are given by (31) and (32), and single real roots are given by the unique solution pair of
  33 $$\Theta = \overline{\alpha }{\text{e}}^{{ - \overline{\alpha }}} ,\overline{\alpha } \in {\mathbb{R}}$$
- If $\Theta = {\text{e}}^{ - 1}$, complex conjugate zeros of $q_{SFOTDM} \left( s \right)$ are given by (31) and (32), and the multiple real root reads $s_{1,2} = - \vartheta^{ - 1}$ (i.e., $\overline{\alpha } = 1$).□

#### Proof

 Consider item a) first. From Theorem 1 and Lemma 6, there are no real solutions of (20). Complex conjugate ones have to satisfy

34 $$\left. {q_{SFOTDM} \left( s \right)} \right|_{{s = \alpha + \omega {\text{j}}}} = 0$$

i.e., both the real and imaginary parts must be equal to zero

35 $$\begin{gathered} T\alpha + {\text{e}}^{ - \vartheta \alpha } \cos \left( {\vartheta \omega } \right) = 0 \hfill \\ T\omega - {\text{e}}^{ - \vartheta \alpha } \sin \left( {\vartheta \omega } \right) = 0 \hfill \\ \end{gathered}$$

After some algebraic manipulation, conditions (35) become

36 $$\begin{gathered} - \overline{\alpha } + \Theta {\text{e}}^{{\overline{\alpha }}} \cos \overline{\omega } = 0 \hfill \\ \overline{\omega } - \Theta {\text{e}}^{{\overline{\alpha }}} \sin \overline{\omega } = 0 \hfill \\ \end{gathered}$$

By expressing $\Theta {\text{e}}^{{\overline{\alpha }}}$ from one equation und substituting it into another one, formula (31) is obtained where singularities are to be denied. Further, the latter equation in (36) gives

37 $${\text{e}}^{{\overline{\alpha }}} = \frac{{\overline{\omega }}}{{\Theta \sin \overline{\omega }}}$$

which yields (32) directly. Naturally, only positive right-hand sides of (37) are admissible to get real $\overline{\alpha }$ values.

We know from Lemmas 5 and 6 and Theorem 1 that the only possible double real root bifurcates into a complex conjugate pair for $\Theta > {\text{e}}^{ - 1}$ and there cannot exist another real root of $q_{SFOTDM} \left( s \right)$. Note that only one root from the pair is sufficient to take due to the symmetry.

Assuming item b), a pair of single real roots exists due to Theorem 1. However, it is the only such a pair according to Lemma 6. A single real root must satisfy the first condition in (26), giving rise to (27), the result of which agrees with (33). However, complex conjugate zeros of $q_{SFOTDM} \left( s \right)$ must simultaneously exist due to its transcendental manner.

Regarding item c), the existence of the double real root is given by Lemma 2. Besides, there is no other real root of $q_{SFOTDM} \left( s \right)$ due to Lemma 5, yet $s_{i} \in {\mathbb{C}}\backslash {\mathbb{R}}$ as in (31) and (32) still exist.■

Theorem 2 does not consider multiple quasi-polynomial roots $s_{i} \in {\mathbb{C}}\backslash {\mathbb{R}}$. The following proposition verifies that such roots can be neglected.

#### Proposition 2

 Equation (20) does not admit a multiple pair solution $s_{i} = \alpha + \omega {\text{j,}}\,\overline{s}_{i} = \alpha - \omega {\text{j}}$,$\omega > 0$. □

#### Proof

Any *n*-multiple $s_{i} \in {\mathbb{C}}\backslash {\mathbb{R}}$ must satisfy (35) and also

38 $$\left. {q^{\left( k \right)}_{SFOTDM} \left( s \right)} \right|_{{s = \alpha + \omega {\text{j}}}} = 0,\,\,k = 1,2,\ldots ,n - 1$$

It is enough to show that a complex conjugate pair of multiplicity 2 does not exist, i.e., $n = 1$. We proof a contradiction; hence, let there exists a double root $s_{i} \in {\mathbb{C}}\backslash {\mathbb{R}}$ that has to satisfy

$$\begin{aligned} \left. {0 = q^{\prime}_{SFOTDM} } \right|_{{s = \alpha + \omega {\text{j}}}} & = \left. {T - \vartheta {\text{e}}^{ - \vartheta } } \right|_{{s = \alpha + \omega {\text{j}}}} \\ & = T - \vartheta {\text{e}}^{ - \alpha \vartheta } \cos \left( {\omega \vartheta } \right) - {\text{j}}\vartheta {\text{e}}^{ - \alpha \vartheta } \sin \left( {\omega \vartheta } \right) \\ & = T - \vartheta {\text{e}}^{{\overline{\alpha }}} \cos \overline{\omega } - {\text{j}}\vartheta {\text{e}}^{{\overline{\alpha }}} \sin \overline{\omega } \\ \end{aligned}$$

which gives

39 $$\begin{array}{*{20}l} {1 - \Theta {\text{e}}^{{\overline{\alpha }}} \cos \overline{\omega } = 0} \hfill \\ {\Theta {\text{e}}^{{\overline{\alpha }}} \sin \overline{\omega } = 0} \hfill \\ \end{array}$$

The latter formula in (39) has two solutions: $\Theta {\text{e}}^{{\overline{\alpha }}} = 0$ or $\sin \overline{\omega } = 0$. The first one is in the contradiction to the former condition in (39), whereas the second one yields

40 $$\sin \overline{\omega } = k\pi ,\,k = 0,1,\ldots \,\,$$

By substituting (40) into the first condition in (39), one gets $\Theta {\text{e}}^{{\overline{\alpha }}} = 1$, which implies $\overline{\omega } = \sin \left( {\overline{\omega }} \right)$ from (36) or (37). That is, the unique solution $\overline{\omega } = 0$ means that the quasi-polynomial root is real, which gives a contradiction.■

#### Corollary 1

- If ${\text{e}}^{ - 1} < \Theta < \frac{\pi }{2}$, the rightmost zeros of $q_{SFOTDM} \left( s \right)$ form a complex conjugate pair $s_{i} = - \frac{{\overline{\alpha }}}{\vartheta } + \frac{{\overline{\omega }}}{\vartheta }{\text{j,}}\,\overline{s}_{i} = - \frac{{\overline{\alpha }}}{\vartheta } - \frac{{\overline{\omega }}}{\vartheta }{\text{j}}$, given by the unique solution of the constrained optimization problem
  41 $$\begin{gathered} \mathop {\min }\limits_{{0 < \overline{\alpha } < 1}} \overline{\alpha } = \frac{{\overline{\omega }}}{{\tan \overline{\omega }}} \hfill \\ {\text{s}}{.}\,{\text{t}}{.: }\overline{\alpha } = \ln \left( {\frac{{\overline{\omega }}}{{\Theta \sin \overline{\omega }}}} \right),\overline{\omega } \in \left( {0,\frac{\pi }{2}} \right) \hfill \\ \end{gathered}$$
- If $\Theta \in \left( {0,{\text{e}}^{ - 1} } \right)$, the rightmost zero of $q_{SFOTDM} \left( s \right)$ is a single real root given by the unique solution of
  42 $$\mathop {\min }\limits_{{0 < \overline{\alpha } < 1}} \overline{\alpha } = \Theta {\text{e}}^{{\overline{\alpha }}}$$
- If $\Theta = {\text{e}}^{ - 1}$, the rightmost zero of $q_{SFOTDM} \left( s \right)$ is the double real root $s_{1,2} = - \frac{1}{\vartheta }$ (i.e., $\overline{\alpha } = 1$).□

#### Proof

Regarding item a), the given range of $\Theta$ yields the rightmost complex solutions of (20) according to Theorem 1. Besides, its abscissa is within the range (24). All complex roots have their loci given by (31) and (32) of Theorem 2.

The only fact remaining to prove item a) is to show that $\overline{\omega } \in \left( {0,0.5\pi } \right)$ if and only if $\overline{\alpha } = \left( {0,1} \right)$ whenever $\Theta \in \left( {{\text{e}}^{1} ,0.5\pi } \right)$. From Lemma 3, $\mathop {\lim }\limits_{{\Theta \to 0.5\pi^{ - } }} \overline{\omega } = 0.5\pi^{ - }$ and $\mathop {\lim }\limits_{{\Theta \to {\text{e}}^{{\left( { - 1} \right) + }} }} \overline{\omega } = 0$ due to Lemma 2 (i.e., the complex pair becomes the double real $q_{SFOTDM} \left( s \right)$ zero).

(Necessity.) Now, we prove by contradiction that $\overline{\omega }$ remains within the limit. Consider that $\overline{\alpha } = \left( {0,1} \right)$. Let exist $\overline{\omega } = 0$ or $\overline{\omega } = \frac{\pi }{2}$ such that the first equation in (41) holds. The limit values are, respectively,

43 $$\begin{gathered} \mathop {\lim }\limits_{{\overline{\omega } \to \frac{\pi }{2}}} \overline{\alpha } = \mathop {\lim }\limits_{{\overline{\omega } \to \frac{\pi }{2}}} \frac{{\overline{\omega }}}{{\tan \overline{\omega }}} = 0 \hfill \\ \mathop {\lim }\limits_{{\overline{\omega } \to 0}} \overline{\alpha } = \mathop {\lim }\limits_{{\overline{\omega } \to 0}} \frac{{\overline{\omega }}}{{\tan \overline{\omega }}} = \mathop {\lim }\limits_{{\overline{\omega } \to 0}} \frac{1}{{\frac{1}{{\cos^{2} \overline{\omega }}}}} = 1 \hfill \\ \end{gathered}$$

which is in contradiction to $\overline{\alpha } = \left( {0,1} \right)$.

The case $\overline{\omega } < 0$ can be omitted due to the root loci symmetry in ${\mathbb{C}}$. Whenever $\overline{\omega } > 0.5\pi$, then there exists $\tan \overline{\omega } < 0$, which implies $\overline{\alpha } < 0$ from (31), and we have a contradiction again.

(Sufficiency). It holds that $\overline{\omega } < \tan \overline{\omega }$ for $\overline{\omega } \in \left( {0,0.5\pi } \right)$, which gives $\overline{\alpha } = \left( {0,1} \right)$ directly.

Regarding item b), there exist two single real zeros of $q_{SFOTDM} \left( s \right)$ due to Lemma 6, the loci of which are given by (33) in Theorem 2. In addition, the right root from the pair determines the spectral abscissa for $\Theta \in \left( {0,{\text{e}}^{ - 1} } \right)$ from Theorem 1, which implies (42).

Item c) represents a reformulation of Lemma 2.■

#### Remark 1

Corollary 1 can be extended to an unstable case; however, it is useless for this study. If needed, Theorem 2 can be generally used. □

**Figure 7:**
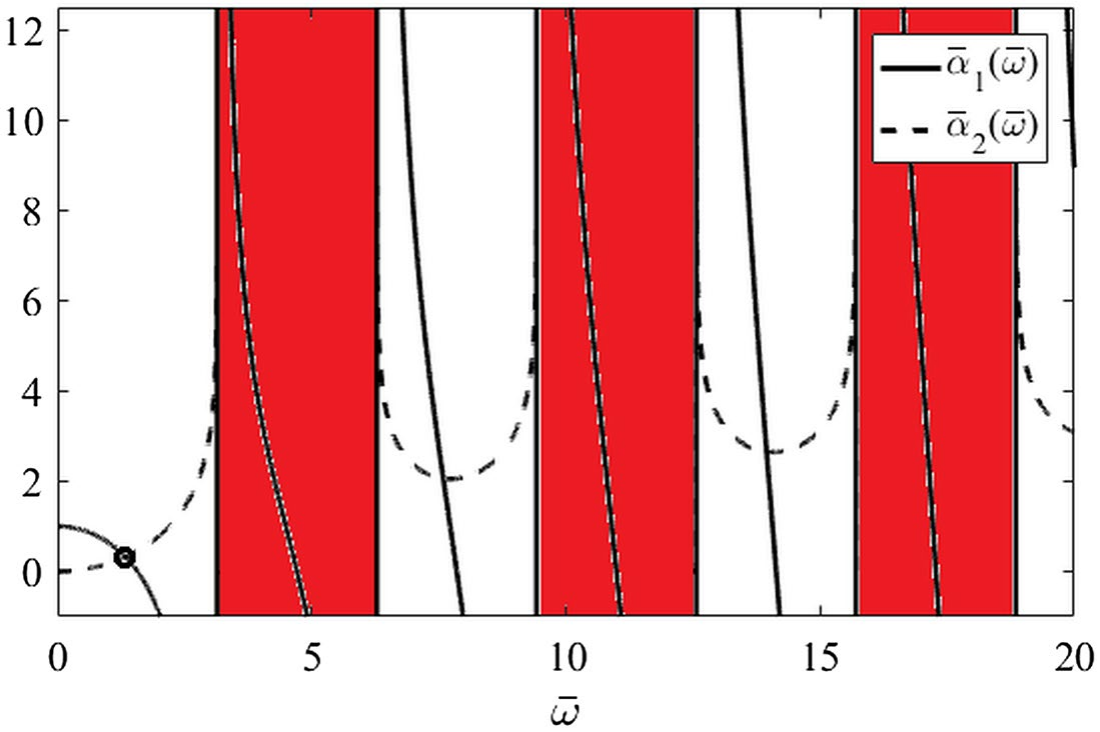
Intersections of $\overline{\alpha }_{1} \left( {\overline{\omega }} \right)$ and $\overline{\alpha }_{2} \left( {\overline{\omega }} \right)$ to get $s_{i} \in {\mathbb{C}}\backslash {\mathbb{R}}$ (Theorem 2, Corollary 1).

To conclude this subsection, a graphical procedure to find all the roots of $q_{SFOTDM} \left( s \right)$ or the rightmost spectrum of the SFOTDM poles follows. Whenever condition of item b) of Theorem 2 is satisfied, real poles are found as per Fig. 6. Complex conjugate poles are given by (31) and (32) or (41), which can be graphically interpreted as intersections of real-valued functions $\overline{\alpha }_{1} \left( {\overline{\omega }} \right) = \overline{\omega }/\tan \overline{\omega }$ and $\overline{\alpha }_{2} \left( {\overline{\omega }} \right) = \ln \left( {\overline{\omega }/\left( {\Theta \sin \overline{\omega }} \right)} \right)$ where values $\overline{\alpha }_{2} \left( {\overline{\omega }} \right) \in {\mathbb{C}}\backslash {\mathbb{R}}$ determine “forbidden regions” of the function graph, see Fig. 7 for illustration.

The example figure is done for $T = \vartheta = \Theta = 1$. Forbidden regions are highlighted in red. The circle indicates the position on the dominant complex conjugate pair according to (41).

### Relay-based parameter identification of heat-exchanger process

#### Infinite-dimensional heat-exchanger process model

**Figure 8:**
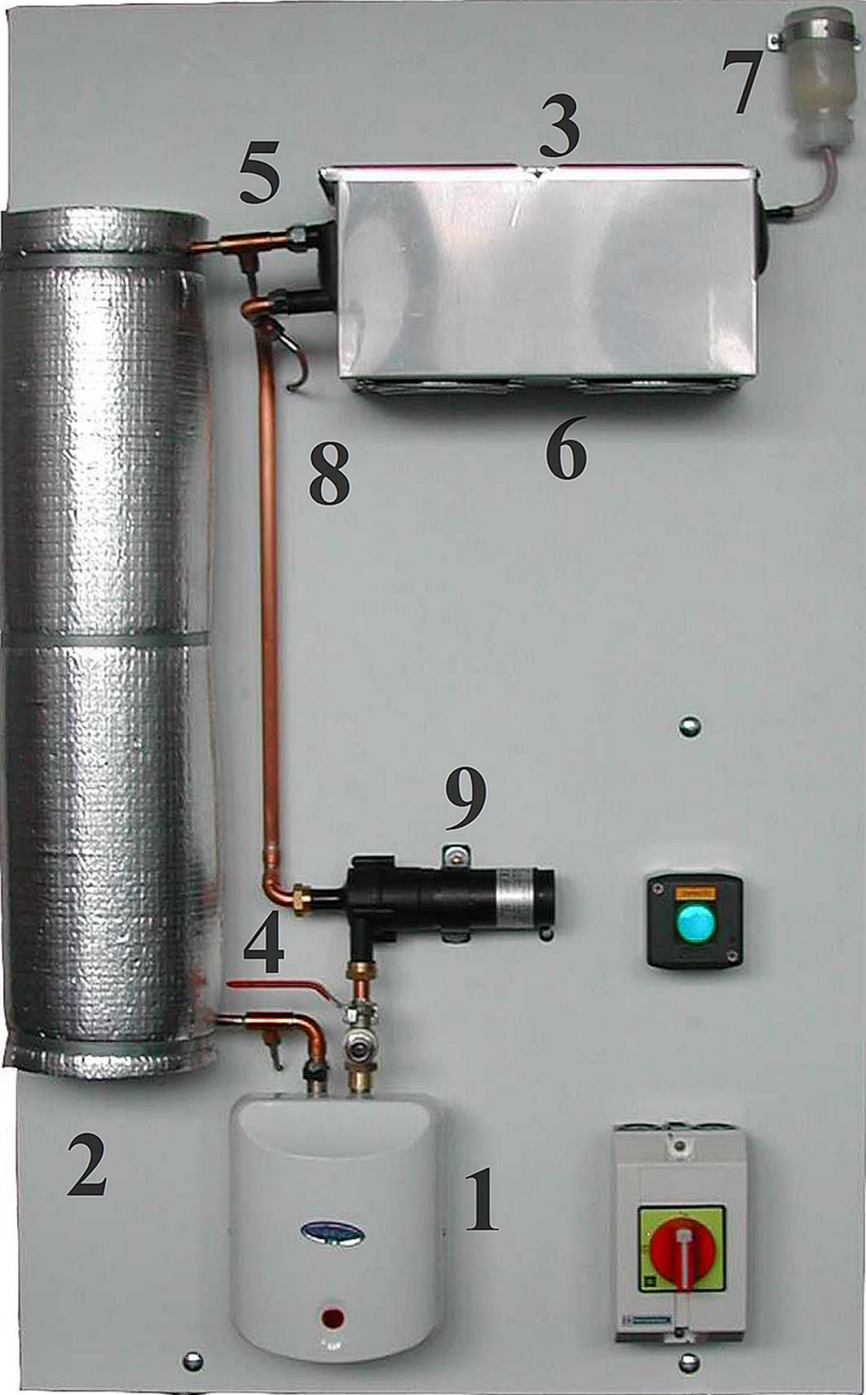
The HETDM appliance.

The HETDM serves as a simulation testbed. The mathematical model arises from heat and mass balance equations that include delays and a thorough analysis of static and dynamic responses of the particular laboratory appliance (see Fig. 8). A concise description of the apparatus follows^84^ first. Positions in the figure correspond to the numbers in curly brackets.

The heat fluid circulates in the closed loop flowing through an instantaneous heater {1}, a long insulated coiled pipeline {2}, and a cooler {3}. The power input to the heater (that can be viewed as a solid–liquid flow heat exchanger) is continuously controlled in the pulse-width-modulation sense. Its maximum value is 750 W. The heated fluid temperature on the heater output {4} is only slightly affected when flowing through the 15 m long pipeline; however, the most significant loop delay is caused therein. The outlet temperature of the pipeline is measured by a platinum resistance Pt1000 thermometer {5}. The cooler is constructed as a radiator (i.e., a plate-and-fin heat exchanger) that can be considered as an indirect unmixed cross-flow heat exchanger from the process point of view. It is equipped with two cooling fans {6} (one of them is continuously controlled, while another is on/off type). The expansion tank compensates for the impact of the water thermal expansion {7}. The outlet temperature from the cooler is measured by Pt1000 again {8}. Finally, the continuously controllable magnetic drive centrifugal pump {9} serves for fluid circulation.

Despite its simplicity, the mathematical formulation of the HETDM and especially its dynamic properties are remarkable due to the model transcendental characteristic equation^84^. As the model is multivariable, the relation between the heater power input $u\left( t \right)$ and the cooler outlet heat fluid temperature $y\left( t \right)$ is selected as the most interesting input–output pair. Note that both quantities are considered as their deviations from a steady state. The analytically modelled linearized relation reads

44 $$y^{\left( 3 \right)} \left( t \right) + a_{2} \ddot{y}\left( t \right) + a_{1} \dot{y}\left( t \right) + a_{0} y\left( t \right) + a_{0\vartheta } y\left( {t - \vartheta } \right) = b_{0} u\left( {t - \tau } \right) + b_{0\tau } u\left( {t - \tau - \tau_{0} } \right)$$

which is a DDE where $b_{0} ,b_{0\tau } ,a_{2} ,a_{1} ,a_{0} ,a_{0\vartheta } \in {\mathbb{R}}$ and $\tau ,\tau_{0} ,\vartheta \in {\mathbb{R}}_{ + }$ express input/output delays and the state (internal) delay, respectively. The corresponding transfer function is

45 $$G_{HETDM} \left( s \right) = \frac{{b_{0} + b_{0\tau } {\text{e}}^{{ - \tau_{0} s}} }}{{s^{3} + a_{2} s^{2} + a_{1} s + a_{0} + a_{0\vartheta } {\text{e}}^{ - \vartheta s} }}{\text{e}}^{ - \tau s}$$

the denominator of which represents the model characteristic RQP (i.e., $q_{HETDM}$). In^84^, the following parameter values have been determined by a thorough and complex analysis of static and dynamic data

46 $$\begin{gathered} a_{2} = 1.{722} \times {1}0^{ - 1} ,a_{1} = 8.509 \times {1}0^{ - 3} ,a_{0} = {1}.298 \times {1}0^{{ - {4}}} ,a_{0\vartheta } = - {7}.0{22} \times {1}0^{{ - {5}}} , \hfill \\ b_{0} = - 2.496 \times {1}0^{{ - {7}}} ,b_{0\tau } = 2.173 \times {1}0^{{ - {6}}} ,\tau = 141,\tau_{0} = 1.5,\vartheta = 151 \hfill \\ \end{gathered}$$

Let us take these data as a benchmark for the significantly more straightforward relay-based experiment. As the values arise from determined physical quantities of the process, they are assumed to be closed to the actual (true) real-life values.

##### Remark 2

 The used Pt1000 thermometers have the guaranteed time constant $T_{63}$ of 8 s, i.e., $T_{90} \approx 18.4$ s. This additional dynamical latency has not been taken into account in analytically-derived model (44), and the true temperature values can be different from the measured ones. However, such negligence does not pose a serious problem with the model. First, plant delays $\tau ,\vartheta$ caused by the thermal fluid transportation have significantly higher values, approx. $\approx 150$ s. This means that possible sensor latencies have only a minor effect on the overall dynamics. Second, sensors' latencies do not affect the internal delay of the model itself since they act only in the input/output relation; yet, they are included in the internal delay of the relay-feedback closed loop. If the system is considered linear (indeed, model (44) is a linearized formulation valid in the vicinity of an operating point), a sensor delay can be considered as the additional input/output delay of the model. As input/output delays are not derived analytically but based on measurements, the relay experiment data’s evaluation also covers these non-modeled latencies. Therefore, once the model is used for plant control, the plant model and the output signal for the feedback have the same value (in the ideal case).

### Simple model parameter estimation using the relay-based experiment

The first step of the identification chain is estimating the SFOTDM parameters, especially those of $q_{SFOTDM}$ (20). It has three substeps. First, the on/off relay with $\delta > 0$ (and $\varepsilon$ being sufficiently small), see (5), is used to estimate the static gain $k$ in (19) as per (6). Second, the ideal relay ($\delta = 0$) is applied to get the initial estimation of oscillation data and the input/output delay value. Finally, the saturation relay is used to improve the accuracy of oscillation parameters, which yields the SFOTDM parameters from (4) and (7). All the substeps can be done within a single experiment, saving time since the transition from particular substantial oscillations to others takes less time than setting the oscillations from a constant steady state.

Let us use the notation $\tau \to \tau_{s} ,\vartheta \to \vartheta_{s}$ for (19) to distinguish the SFOTDM parameters from those of the HETDM (for which no subscript is used). The combination of (4) and (7) can be solved analytically yielding^12^

47 $$\,T = \frac{{\sin \left( {\omega_{osc} \vartheta_{s} } \right) - \tan \left( {\omega_{osc} \tau_{s} } \right)\cos \left( {\omega_{osc} \vartheta_{s} } \right)}}{{\omega_{osc} }},\,\,\vartheta_{s} = \frac{{\pi - \cos^{ - 1} \left( {kN_{ \cdot } \left( {A, \cdot } \right)\cos \left( {\omega_{osc} \tau_{s} } \right)} \right)}}{{\omega_{osc} }}$$

where $N_{ \cdot } \left( {A, \cdot } \right)$ stands for either (5) or (7). However, it is inherently expected that the argument of $\cos^{ - 1} \left( \cdot \right)$ is within the range $\left[ { - 1,1} \right]$. Whenever it does not hold, a numerical solution of the combination of (4) and (7) have to be used instead of (47).

**Figure 9:**
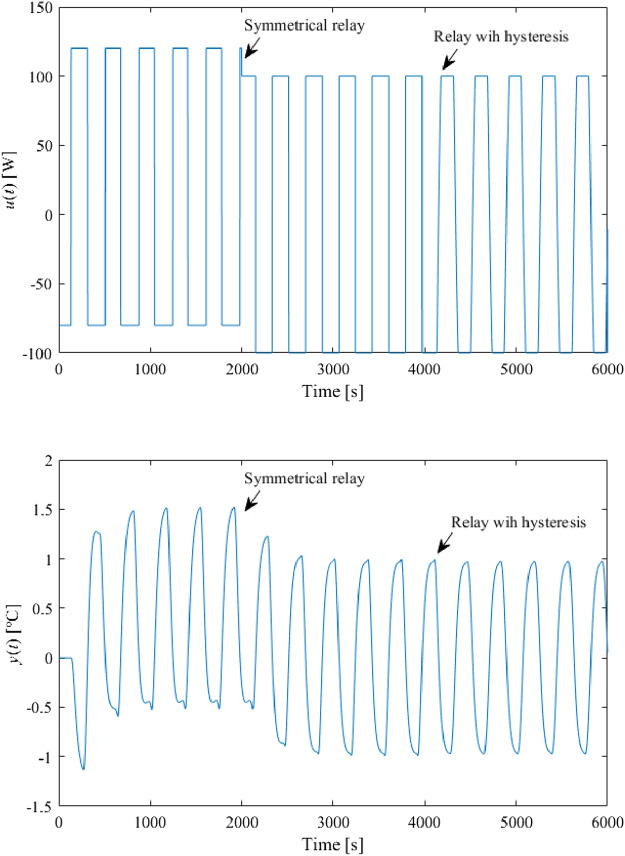
Relay experiment responses (without an artificial delay).

Set $B = 100$, $\delta = 0.05$, and $\varepsilon = 10^{ - 5}$. The relay-test responses are displayed in Fig. 9. The arrows indicate when a particular relay starts to be used.

The eventual data from Fig. 9 are summarized in Table 1. Formula (19) gives $k = 3.22 \times 10^{ - 2}$. The value of $\tau_{s}$ can be estimated as the time interval between the switching point of $u\left( t \right)$ and the peak time instant of $y\left( t \right)$. Hence, it can be measured that $\tau_{s} \approx 136.7$ s. Note that $k_{\min } = 1.4$ has been taken for the saturation relay setting, which gives rise to $k_{sat} = 185.1$, $\overline{A} = 0.555$.

**Table 1:** Relay experiment data (without an artificial delay).

Relay used	$A$(°C)	$N_{ \cdot } \left( {A, \cdot } \right)$(W/°C)	$T_{osc}$(s)	$\omega_{osc}$(rad/s)
On/off	0.989	128.68	363.9	1.727 × 10^−2^
Saturated	0.971	123.59	369.7	1.700 × 10^−2^

As $kN_{ \cdot } \left( {A, \cdot } \right)\cos \left( {\omega_{osc} \tau_{s} } \right) = - 2.72$ (for the relay with saturation), (47) cannot be used. Hence, we attempt to apply the NM method to solve the minimization problem

48 $$\begin{gathered} \left[ {T,\vartheta_{s} ,\tau_{s} } \right]^{*} = \arg \min f\left( {T,\vartheta_{s} ,\tau_{s} } \right) \hfill \\ f\left( {T,\vartheta_{s} ,\tau_{s} } \right): = \,\left( {\left| {\left. {G_{o,SFOTDM} \left( s \right)} \right|_{{s = {\text{j}}\omega_{osc} }} } \right| - 1} \right)^{2} + \left( {\measuredangle \left( {\left. {G_{o,SFOTDM} \left( s \right)} \right|_{{s = {\text{j}}\omega_{osc} }} } \right) + \pi } \right)^{2} \hfill \\ G_{o,SFOTDM} \left( s \right) = N_{sat} \left( {A,\overline{A}} \right)\left. {G_{SFOTDM} \left( s \right)} \right| \hfill \\ {\text{s}}{.}\,{\text{t}}{.}: - \left[ {T,\vartheta_{s} ,\tau_{s} } \right] < 0 \hfill \\ \end{gathered}$$

where $G_{SFOTDM} \left( s \right)$ is given (19), $N_{sat} \left( {A,\overline{A}} \right)$ and $\omega_{osc}$ are taken from Table 1. The barrier function is chosen as $f_{b} \left( {T,\vartheta_{s} ,\tau_{s} } \right) = - \sum\nolimits_{{x \in \left\{ {T,\vartheta_{s} ,\tau_{s} } \right\}}}^{{}} {\ln \left( {1 - {\text{e}}^{ - x} } \right)}$. The NM control parameters are set to $\,\,\gamma_{r} = 1$, $\,\,\gamma_{e} = 2$, $\,\,\gamma_{oc} = \gamma_{ic} = \gamma_{s} = 0.5$. The initial estimation reads

49 $${}^{1}T = \,\,\frac{1}{{\omega_{osc} }},\,{}^{1}\vartheta_{s} = \frac{\pi T}{4} = \frac{\pi }{{4\omega_{osc} }}$$

The setting of ${}^{1}T$ in (49) arises from the assumption that the inverse of $\omega_{osc} \approx \omega_{u}$ is close to the time constant of the delay-free system. The value of ${}^{1}\vartheta_{s}$ represents the mid-point of the stability interval (21). Two scenarios for many setting combinations of the initial simplex size and $\beta$ in (18) are made. First, the fixed value $\tau_{s} = 136.7$ is assumed (i.e., only $T,\vartheta_{s}$ are optimized). Second, all three parameters in (48) are to be found.

**Figure 10:**
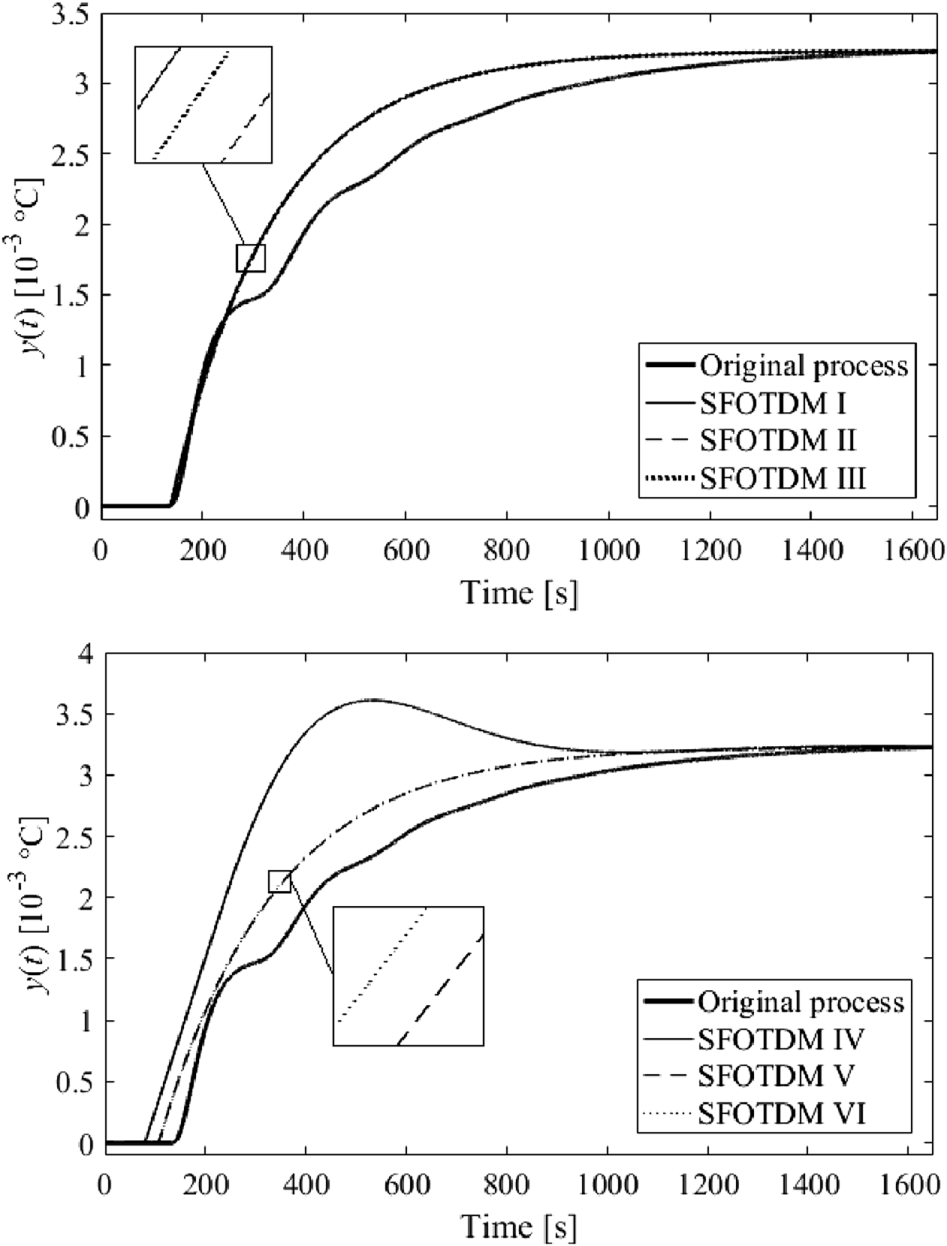
Comparison of SFOTDM unit step responses (vs. the original process response).

Among dozens of results (models), six of the most distinguished ones are summarized in Table 2. The results either minimize $f\left( {T,\vartheta_{s} ,\tau_{s} } \right)$ in the frequency domain or integral absolute error (IAE) and integral time absolute error (ITAE) criteria in the time domain, or represent a trade-off of all the criteria values. Unit step responses (i.e., $u = 1$ W) are displayed in Fig. 10. The models in Table 2 are also equipped with Roman numerals to label them.

**Table 2:** SFOTDM parameter identification results.

Result #	$T$(s)	$\vartheta_{s}$(s)	$\tau_{s}$(s)	$f\left( {T^{*} ,\vartheta_{s}^{*} ,\tau_{s}^{*} } \right)$	IAE	ITAE
I	202.937	0.592	136.7	0.237	313.7	2.248 × 10^5^
II	229.612	25.110	136.7	0.263	309.5	2.226 × 10^5^
III	219.654	16.385	136.7	0.248	311.9	2.239 × 10^5^
IV	258.617	155.647	79.377	2.99 × 10^–27^	812.8	4.432 × 10^5^
V	239.437	9.2304	106.869	1.74 × 10^–5^	314.2	2.067 × 10^5^
VI	247.880	18.421	106.390	8.81 × 10^–10^	318.2	2.090 × 10^5^

**Figure 11:**
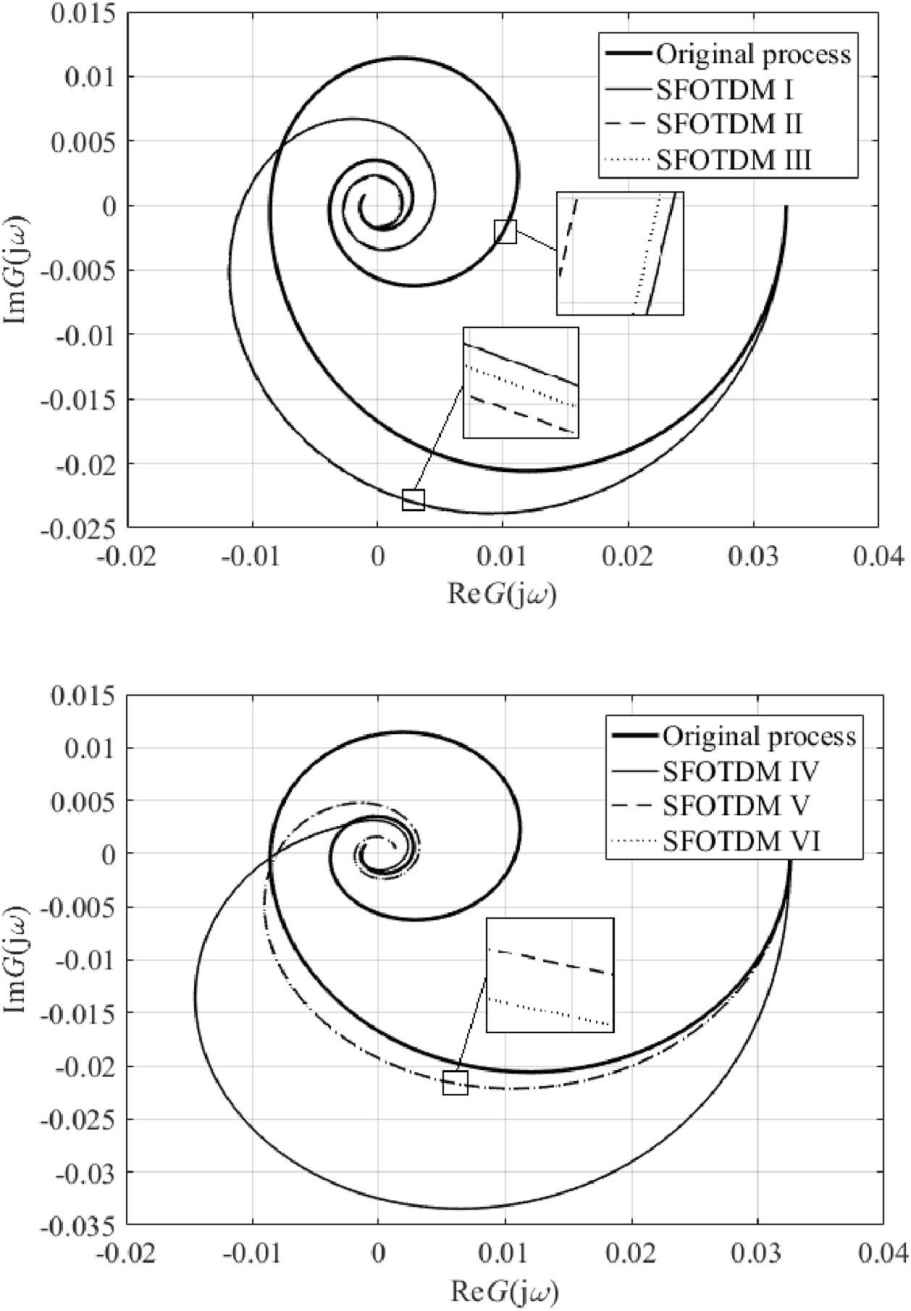
Comparison of SFOTDM Nyquist plots (vs. the original process characteristics).

Results IV, V, and VI provide an outstanding cost function value in the frequency domain, yet a worse IAE criterion compared to the remaining results. However, results V and VI give the best ITAE value. Unsatisfactory time-domain response of parameters IV can be due to an excellent characterization of the ultimate point $G_{SFOTDM} \left( {{\text{j}}\omega_{u} } \right) = - N_{sat}^{ - 1} \left( {A,\overline{A}} \right)$ but an erroneous estimation of the remaining Nyquist plot. Hence, we do let provide the reader with graphical comparisons of process and model Nyquist plots, see Fig. 11.

Note that the terminal frequency value in Fig. 11 is $\omega_{fin} = 0.1$ rad/s. As can be seen, time and frequency responses for models I, II, and III, and those for models V and VI almost coincide. Figure 11 also indicates that the fixed input/output delay value yields a worse estimation of the ultimate point (i.e., the model crossing point with the negative real axis is quite far from that of the original process characteristics). Moreover, results V and VI seem to give the closest frequency-domain responses to the original Nyquist plot. Hence, Table 3 provides root means squares (RMS) values to measure the error between the process and the models. Two terminal frequency values for RMS computation are chosen, $\omega_{fin} = 0.1$ and $\omega_{fin} = \omega_{osc} = {1.7}\times{1}0^{{ - {2}}}$ rad/s.

**Table 3:** SFOTDM Nyquist plot RMS errors.

SFOTDM #	I	II	III	IV	V	VI
RMS ($\times 10^{ - 3}$)$\omega_{fin} = 0.1$	2.639	2.611	2.621	5.545	3.852	3.846
RMS ($\times 10^{ - 3}$)$\omega_{fin} = 1.7 \times 10^{ - 2}$	3.272	3.288	3.279	9.078	2.378	2.419

Data in Table 3 prove the above-introduced assumption that Models V and VI estimate the process frequency response reasonably on low frequencies (i.e., up to the oscillation frequency); however, they fail for higher ones. It is also worth noting that the estimation of the ultimate frequency is quite accurate since the true one is about $\omega_{u} = 1.67 \times 10^{ - 2}$ rad/s.

### Parameter estimation of the heat-exchanger process model via pole assignment

Now, models (results) in Table 2 serve as the initial estimates for HETDM parameters identification. Several scenarios are tested and compared after the HETDM dominant spectrum assignment according to the pole loci of the SFOTDM.

#### Initial denominator parameters estimation

The rightmost spectra of SFOTDMs (see Table 2) poles are displayed in Table 4. These loci are computed via the technique presented in “Root loci analysis of the simple quasi-polynomial”. From Table 4, it can be deduced that the rightmost pole has a decisive impact on the dynamic properties (by comparing the result for SFOTDM I, II, and III), since although the remaining spectra significantly differ, the model time is the same and frequency domain responses are almost identical. Besides, by comparing spectra of models III and VI that are very close to each other, different dynamic properties indicate a high impact of the value of $\tau_{s}$.

**Table 4:** Dominant pole SFOTDMs spectra.

SFOTDM #	Spectrum $\Sigma_{SFOTDM} = \left\{ {s_{1} ,s_{2} ,\ldots } \right\}$
I	$- 4.942 \times 10^{ - 3} , - 13.3446, \ldots$
II	$$\begin{gathered} - 4.929 \times 10^{ - 3} , - 1.3748 \times 10^{ - 1} ,\left( { - 1.7344 \pm 2.9141{\text{j}}} \right) \times 10^{ - 1} , \hfill \\ \left( { - 1.9502 \pm 5.4943{\text{j}}} \right) \times 10^{ - 1} , \ldots \hfill \\ \end{gathered}$$
III	$$\begin{gathered} - 4.936 \times 10^{ - 3} , - 2.4265 \times 10^{ - 1} ,\left( { - 2.9039 \pm 4.4398{\text{j}}} \right) \times 10^{ - 1} , \hfill \\ \left( { - 3.2267 \pm 8.4042{\text{j}}} \right) \times 10^{ - 1} , \ldots \hfill \\ \end{gathered}$$
IV	$$\begin{gathered} \left( { - 4.293 \pm 6.202} \right) \times 10^{ - 3} ,\left( { - 1.6585 \pm 4.8337{\text{j}}} \right) \times 10^{ - 2} , \hfill \\ \left( { - 2.0340 \pm 8.9391{\text{j}}} \right) \times 10^{ - 2} ,\left( { - 2.2685 \pm 13.088{\text{j}}} \right) \times 10^{ - 2} , \ldots \hfill \\ \end{gathered}$$
V	$$\begin{gathered} - 4.347 \times 10^{ - 3} , - 5.2336 \times 10^{ - 1} ,\left( { - 5.9124 \pm 7.8066{\text{j}}} \right) \times 10^{ - 1} , \hfill \\ \left( { - 6.4586 \pm 14.8720{\text{j}}} \right) \times 10^{ - 1} , \ldots \hfill \\ \end{gathered}$$
VI	$$\begin{gathered} - 4.373 \times 10^{ - 3} , - 2.1612 \times 10^{ - 1} ,\left( { - 2.5852 \pm 3.9490{\text{j}}} \right) \times 10^{ - 1} , \hfill \\ \left( { - 2.8722 \pm 7.4755{\text{j}}} \right) \times 10^{ - 1} , \ldots \hfill \\ \end{gathered}$$

The pole assignment problem can be characterized by the set of nonlinear algebraic equations to be solved

50 $$\left. {\left. {{\text{CE}}_{HETDM} \left( {s,a_{2} ,a_{1} ,a_{0} ,a_{0\vartheta } ,\vartheta } \right)} \right|_{{s = \left\{ {s_{1} ,s_{2} ,\ldots } \right\} \in \Sigma_{SFOTDM} }} :q_{HETDM} \left( {s,a_{2} ,a_{1} ,a_{0} ,a_{0\vartheta } ,\vartheta } \right) = 0} \right|_{{s = \left\{ {s_{1} ,s_{2} ,\ldots } \right\} \in \Sigma_{SFOTDM} }}$$

Since problem (50) includes five unknown parameters to be determined, the unique solution (under the assumption that the Jacobian of the $q_{HETDM}$ has the full rank) requires taking $\left\{ {s_{1} ,s_{2} ,s_{3} ,s_{4} ,s_{5} } \right\}$. However, it is not always possible. For instance, SFOTDM I has only two significant real poles (the rest are too far in the LHP), and complex conjugate pairs of other spectra cannot be decoupled. Hence, only less number than five of SFOTDM poles are taken in (50). It is also worth noting that whenever $s_{k} \in {\mathbb{C}}\backslash {\mathbb{R}}$, the $q_{HETDM}$ in (50) is split into its real and imaginary parts.

We use the LM method (see “Levenberg–Marquardt method”) for solving the pole assignment problem. Let us discuss the ideal initial parameters’ selection, ${}^{1}{\mathbf{p}} = {}^{1}\left( {a_{2} ,a_{1} ,a_{0} ,a_{0\vartheta } ,\vartheta } \right)^{T}$. Take the ${\text{CE}}_{HETDM}$ and divide both the sides by $a_{0\vartheta }$

51 $$\frac{1}{{a_{0\vartheta } }}s^{3} + \frac{{a_{2} }}{{a_{0\vartheta } }}s^{2} + \frac{{a_{1} }}{{a_{0\vartheta } }}s + \frac{{a_{0} }}{{a_{0\vartheta } }} + {\text{e}}^{ - \vartheta s} = 0$$

Then, it is apparent that the setting

52 $$a_{2} = a_{0} \to 0,\,\,a_{1} = a_{0\vartheta } T,\,\,\,a_{0\vartheta } \to \infty ,\,\,\vartheta = \vartheta_{s}$$

yields the ${\text{CE}}_{SFOTDM}$. However, such a solution is non-feasible and may result in an immature solution of (50). Therefore, several approximate feasible initial settings are eventually chosen.

The multiplicative parameter $\kappa$ that alternates the damping factor $\lambda$ in iteration steps should increase $\lambda$ when the residual sum on the right-hand side of (13) increases and vice versa. Some authors suggest setting an asymmetric $\lambda$^97^. Hence, after some numerical experiments, we eventually set $\kappa = 5$ when stepping up and $\kappa = 10$ when stepping down.

Due to numerous settings of ${}^{1}\lambda ,{}^{1}{\mathbf{p}}$, a bunch of possible results is obtained. Selected results (i.e., $q_{HETDM}$ parameter values) and their residual sums ${\text{Res}}\left( {{\mathbf{p}}_{opt} } \right)$ (see (13)) are provided to the reader in Table 5. Corresponding dominant pole loci of the models are enumerated in Table 6. Note that the QPmR software package^18^ is utilized to compute the poles here.

**Table 5:** HETDM characteristic RQP parameters after pole assignment.

$q_{HETDM}$ #	$a_{2}$	$a_{1}$	$a_{0}$	$a_{0\vartheta }$	$\vartheta$(s)	${\text{Res}}\left( {{\mathbf{p}}_{opt} } \right)$
I-a	1	2.0294 × 10^4^	0.5003	99.4991	0.5933	4.923 × 10^–9^
I-b	0.1003	202.9372	4.8135 × 10^–2^	0.9517	0.6429	4.079 × 10^–9^
I-c	1	2.0294 × 10^3^	0.5010	9.4998	0.6020	4.855 × 10^–9^
I-d	0.1	2.0294 × 10^3^	4.9865 × 10^–2^	9.9498	0.5989	6.338 × 10^–9^
II-a	3.3354 × 10^3^	3.5842 × 10^3^	− 1.0924	16.5911	24.0343	7.252 × 10^–7^
II-b	69.1007	2.2958 × 10^3^	2.8047 × 10^–5^	9.9987	25.0797	3.531 × 10^–9^
II-c	333.0099	356.8123	− 0.1088	1.6517	24.0316	6.774 × 10^–7^
II-d	32.9795	34.3082	− 0.0105	0.1588	24.0036	9.476 × 10^–8^
III-a	65.8610	2.2007 × 10^3^	− 3.0638 × 10^–4^	10.0189	16.3554	4.291 × 10^–9^
III-b	1.7687	1.2524	− 2.0196 × 10^–4^	5.8940 × 10^–3^	14.8082	8.099 × 10^–7^
III-c	1.7199	2.1965 × 10^4^	2.0309 × 10^–2^	99.9985	16.3852	7.866 × 10^–8^
III-d	66.9478	2.1995 × 10^3^	− 3.2145 × 10^–4^	10.0138	16.3548	5.010 × 10^–9^
III-e	21.3333	221.6120	− 4.2925 × 10^–5^	1.0089	16.2890	4.502 × 10^–9^
III-f	1.7964	1.2943	− 1.9689 × 10^–4^	6.0801 × 10^–3^	14.8407	3.408 × 10^–8^
IV-a	0.2044	63.8564	− 5.6594 × 10^–6^	0.2469	155.6432	6.165 × × 10^–7^
IV-b	1.8438	2.4484 × 10^4^	− 6.3932 × 10^–3^	94.6770	155.6457	1.237 × 10^–8^
IV-c	1.4218	1.0081	− 1.5547 × 10^–7^	3.8981 × 10^–3^	154.2347	7.703 × 10^–7^
IV-d	0.1054	64.5774	− 5.8458 × 10^–6^	0.2497	155.6448	5.002 × 10^–7^
V-a	1.6308	1.0445	− 3.4296 × 10^–3^	7.7139 × 10^–3^	6.6212	8.527 × 10^–7^
V-b	2.2298	1.8530	− 1.5718 × 10^–3^	9.2720 × 10^–3^	7.6344	2.249 × 10^–7^
V-c	39.7452	2.3944 × 10^4^	0.1101	99.9959	9.2288	9.011 × 10^–9^
VI-a	65.2071	2.4823 × 10^3^	9.7148 × 10^–4^	10.0141	18.3944	1.507 × 10^–8^
VI-b	0.4771	2.4787 × 10^4^	2.0034 × 10^–3^	99.9955	18.4207	8.334 × 10^–9^
VI-c	8.6334	2.4788 × 10^4^	1.9849 × 10^–3^	99.9988	18.4204	2.004 × 10^–8^
VI-d	0.8673	2.4786 × 10^4^	2.0039 × 10^–3^	99.9922	18.4207	7.501 × 10^–9^
VI-e	65.1724	2.4791 × 10^3^	9.7024 × 10^–4^	10.0012	18.3944	9.421 × 10^–8^
VI-f	22.4011	249.1720	9.1230 × 10^–5^	1.0052	18.3308	6.733 × 10^–9^

**Table 6:** Dominant pole HETDM spectra.

$q_{HETDM}$ #	Spectrum $\Sigma_{HETDM} = \left\{ {s_{1} ,s_{2} ,\ldots } \right\}$
I-a	$- 4.942 \times 10^{ - 3} , - 13.3446,\ldots$
I-b	$- 4.942 \times 10^{ - 3} , - 13.3446,\ldots$
I-c	$- 4.942 \times 10^{ - 3} , - 13.3446,\ldots$
I-d	$- 4.942 \times 10^{ - 3} , - 13.3446,\ldots$
II-a	$$\begin{gathered} - 4.929 \times 10^{ - 3} , - 1.3481 \times 10^{ - 1} ,\left( { - 1.7344 \pm 2.9140{\text{j}}} \right) \times 10^{ - 1} , \hfill \\ \left( { - 1.9978 \pm 5.5034{\text{j}}} \right) \times 10^{ - 1} ,\ldots \hfill \\ \end{gathered}$$
II-b	$$\begin{gathered} - 4.929 \times 10^{ - 3} , - 1.3748 \times 10^{ - 1} ,\left( { - 1.7344 \pm 2.9141{\text{j}}} \right) \times 10^{ - 1} , \hfill \\ \left( { - 1.9502 \pm 5.4943{\text{j}}} \right) \times 10^{ - 1} ,\ldots \hfill \\ \end{gathered}$$
II-c	$$\begin{gathered} - 4.929 \times 10^{ - 3} , - 1.3481 \times 10^{ - 1} ,\left( { - 1.7344 \pm 2.9140{\text{j}}} \right) \times 10^{ - 1} , \hfill \\ \left( { - 1.9978 \pm 5.5034{\text{j}}} \right) \times 10^{ - 1} ,\ldots \hfill \\ \end{gathered}$$
II-d	$$\begin{gathered} - 4.929 \times 10^{ - 3} , - 1.3481 \times 10^{ - 1} ,\left( { - 1.7344 \pm 2.9141{\text{j}}} \right) \times 10^{ - 1} , \hfill \\ \left( { - 1.9978 \pm 5.5034{\text{j}}} \right) \times 10^{ - 1} ,\ldots \hfill \\ \end{gathered}$$
III-a	$$\begin{gathered} - 4.936 \times 10^{ - 3} , - 2.4265 \times 10^{ - 1} ,\left( { - 2.9039 \pm 4.4398{\text{j}}} \right) \times 10^{ - 1} , \hfill \\ \left( { - 3.2266 \pm 8.4042{\text{j}}} \right) \times 10^{ - 1} ,\ldots \hfill \\ \end{gathered}$$
III-b	$$\begin{gathered} - 4.936 \times 10^{ - 3} , - 2.4265 \times 10^{ - 1} ,\left( { - 2.9039 \pm 4.4398{\text{j}}} \right) \times 10^{ - 1} , \hfill \\ \left( { - 3.3332 \pm 8.2918{\text{j}}} \right) \times 10^{ - 1} ,\ldots \hfill \\ \end{gathered}$$
III-c	$$\begin{gathered} - 4.937 \times 10^{ - 3} , - 2.4265 \times 10^{ - 1} ,\left( { - 2.9039 \pm 4.4398{\text{j}}} \right) \times 10^{ - 1} , \hfill \\ \left( { - 3.2266 \pm 8.4042{\text{j}}} \right) \times 10^{ - 1} ,\ldots \hfill \\ \end{gathered}$$
III-d	$$\begin{gathered} - 4.936 \times 10^{ - 3} , - 2.4265 \times 10^{ - 1} ,\left( { - 2.9039 \pm 4.4398{\text{j}}} \right) \times 10^{ - 1} , \hfill \\ \left( { - 3.2266 \pm 8.4042{\text{j}}} \right) \times 10^{ - 1} ,\ldots \hfill \\ \end{gathered}$$
III-e	$$\begin{gathered} - 4.936 \times 10^{ - 3} , - 2.4265 \times 10^{ - 1} ,\left( { - 2.9039 \pm 4.4398{\text{j}}} \right) \times 10^{ - 1} , \hfill \\ \left( { - 3.2266 \pm 8.4042{\text{j}}} \right) \times 10^{ - 1} ,\ldots \hfill \\ \end{gathered}$$
III-f	$$\begin{gathered} - 4.936 \times 10^{ - 3} , - 2.4265 \times 10^{ - 1} ,\left( { - 2.9039 \pm 4.4398{\text{j}}} \right) \times 10^{ - 1} , \hfill \\ \left( { - 3.3325 \pm 8.2958{\text{j}}} \right) \times 10^{ - 1} ,\ldots \hfill \\ \end{gathered}$$
IV-a	$$\begin{gathered} \left( { - 4.293 \pm 6.202{\text{j}}} \right) \times 10^{ - 3} ,\left( { - 1.6585 \pm 4.8337{\text{j}}} \right) \times 10^{ - 2} , \hfill \\ \left( { - 2.0339 \pm 8.9392{\text{j}}} \right) \times 10^{ - 2} ,\left( { - 2.{2684} \pm 1{3}{\text{.0089j}}} \right) \times 10^{ - 2} \hfill \\ \end{gathered}$$
IV-b	$$\begin{gathered} \left( { - 4.293 \pm 6.202{\text{j}}} \right) \times 10^{ - 3} ,\left( { - 1.6585 \pm 4.8337{\text{j}}} \right) \times 10^{ - 2} , \hfill \\ \left( { - 2.0340 \pm 8.9392{\text{j}}} \right) \times 10^{ - 2} ,\left( { - 2.{2684} \pm 1{3}{\text{.0088j}}} \right) \times 10^{ - 2} \hfill \\ \end{gathered}$$
IV-c	$$\begin{gathered} \left( { - 4.293 \pm 6.202{\text{j}}} \right) \times 10^{ - 3} ,\left( { - 1.6585 \pm 4.8337{\text{j}}} \right) \times 10^{ - 2} , \hfill \\ \left( { - 2.0339 \pm 8.9390{\text{j}}} \right) \times 10^{ - 2} ,\left( { - 2.{2682} \pm 1{3}{\text{.0082j}}} \right) \times 10^{ - 2} \hfill \\ \end{gathered}$$
IV-d	$$\begin{gathered} \left( { - 4.293 \pm 6.202{\text{j}}} \right) \times 10^{ - 3} ,\left( { - 1.6585 \pm 4.8337{\text{j}}} \right) \times 10^{ - 2} , \hfill \\ \left( { - 2.0339 \pm 8.9392{\text{j}}} \right) \times 10^{ - 2} ,\left( { - 2.{2683} \pm 1{3}{\text{.0089j}}} \right) \times 10^{ - 2} \hfill \\ \end{gathered}$$
V-a	$$\begin{gathered} - 4.347 \times 10^{ - 3} , - 5.2336 \times 10^{ - 1} ,\left( { - 5.9124 \pm 7.8066{\text{j}}} \right) \times 10^{ - 1} , \hfill \\ \left( { - 9.3259 \pm 15.5209{\text{j}}} \right) \times 10^{ - 1} ,\ldots \hfill \\ \end{gathered}$$
V-b	$$\begin{gathered} - 4.347 \times 10^{ - 3} , - 5.2336 \times 10^{ - 1} ,\left( { - 5.9124 \pm 7.8066{\text{j}}} \right) \times 10^{ - 1} , \hfill \\ \left( { - 7.5211 \pm 14.6316{\text{j}}} \right) \times 10^{ - 1} ,\ldots \hfill \\ \end{gathered}$$
V-c	$$\begin{gathered} - 4.352 \times 10^{ - 3} , - 5.2336 \times 10^{ - 1} ,\left( { - 5.9124 \pm 7.8066{\text{j}}} \right) \times 10^{ - 1} , \hfill \\ \left( { - 6.4586 \pm 14.8720{\text{j}}} \right) \times 10^{ - 1} ,\ldots \hfill \\ \end{gathered}$$
VI-a	$$\begin{gathered} - 4.373 \times 10^{ - 3} , - 2.1612 \times 10^{ - 1} ,\left( { - 2.5852 \pm 3.9490{\text{j}}} \right) \times 10^{ - 1} , \hfill \\ \left( { - 2.8722 \pm 7.4755{\text{j}}} \right) \times 10^{ - 1} ,\ldots \hfill \\ \end{gathered}$$
VI-b	$$\begin{gathered} - 4.373 \times 10^{ - 3} , - 2.1612 \times 10^{ - 1} ,\left( { - 2.5852 \pm 3.9490{\text{j}}} \right) \times 10^{ - 1} , \hfill \\ \left( { - 2.8722 \pm 7.4755{\text{j}}} \right) \times 10^{ - 1} ,\ldots \hfill \\ \end{gathered}$$
VI-c	$$\begin{gathered} - 4.373 \times 10^{ - 3} , - 2.1612 \times 10^{ - 1} ,\left( { - 2.5852 \pm 3.9490{\text{j}}} \right) \times 10^{ - 1} , \hfill \\ \left( { - 2.8722 \pm 7.4755{\text{j}}} \right) \times 10^{ - 1} ,\ldots \hfill \\ \end{gathered}$$
VI-d	$$\begin{gathered} - 4.373 \times 10^{ - 3} , - 2.1612 \times 10^{ - 1} ,\left( { - 2.5852 \pm 3.9490{\text{j}}} \right) \times 10^{ - 1} , \hfill \\ \left( { - 2.8722 \pm 7.4755{\text{j}}} \right) \times 10^{ - 1} ,\ldots \hfill \\ \end{gathered}$$
VI-e	$$\begin{gathered} - 4.373 \times 10^{ - 3} , - 2.1612 \times 10^{ - 1} ,\left( { - 2.5852 \pm 3.9490{\text{j}}} \right) \times 10^{ - 1} , \hfill \\ \left( { - 2.8722 \pm 7.4755{\text{j}}} \right) \times 10^{ - 1} ,\ldots \hfill \\ \end{gathered}$$
VI-f	$$\begin{gathered} - 4.373 \times 10^{ - 3} , - 2.1612 \times 10^{ - 1} ,\left( { - 2.5852 \pm 3.9490{\text{j}}} \right) \times 10^{ - 1} , \hfill \\ \left( { - 2.8722 \pm 7.4755{\text{j}}} \right) \times 10^{ - 1} ,\ldots \hfill \\ \end{gathered}$$

It can be deduced that despite diverse results within each of six models (characteristic RQP) families (i.e., I to VI), the obtained dominant spectra are very close to each other. This yields multimodality of the optimization problem. Characteristic RQPs I-a to I-d, II-b, III-a, III-d, III-e, IV-b, V-c, and VI-a to VI-f give almost identical spectra to the original ones (i.e., those of SFOTDMs). These findings correspond to the values of ${\text{Res}}\left( {{\mathbf{p}}_{opt} } \right)$ in Table 5. It is exciting to observe that even if a subset of two or four poles is prescribed, other one or two complex conjugate pairs coincide with the original spectrum. This feature proves the success of the assignment task and an excellent mapping between $q_{SFOTDM}$ and $q_{HETDM}$ parameters.

Now three different scenarios of how to set numerator parameters of the model transfer function (45) or even eventually alter all the transfer function parameters based on relay-experiment data follow.

#### Numerator parameters estimation using Levenberg–Marquardt method

The first scenario adopts the LM method and utilizes data solely from the single relay test (i.e., without a necessity to perform additional experiments) that should indicate the ultimate data (i.e., the critical point of the Nyquist curve). Once the parameters are determined as in “Initial denominator parameters estimation”, they are fixed. Hence, the HETDM transfer function numerator parameters are estimated to comply with conditions (4). The advantage of this scenario lies in fact that all the model parameters are found within a single test.

In more detail, the set of nonlinear algebraic equations to be solved reads

53 $$\begin{gathered} {\text{Re}} \left. {G_{o,HETDM} \left( {s,b_{0} ,b_{0\tau } ,\tau_{0} ,\tau } \right)} \right|_{{s = {\text{j}}\omega_{osc} }} + 1 = 0,\,{\text{Im}} \left. {G_{o,HETDM} \left( {s,b_{0} ,b_{0\tau } ,\tau_{0} ,\tau } \right)} \right|_{{s = {\text{j}}\omega_{osc} }} = 0 \hfill \\ G_{o,HETDM} \left( {s, \cdot } \right) = N_{sat} \left( {A,\overline{A}} \right)G_{HETDM} \left( {s, \cdot } \right) \hfill \\ \end{gathered}$$

where $\omega_{osc}$ holds when using the relay with saturation. As only two parameters can be determined by solving (53), two other ones need to be set a priori. Let $\tau = \tau_{s}$ be fixed as in Table 2. As the static gain $k = 3.22 \times 10^{ - 2}$ is known, the following equality is substituted to (53)

54 $$b_{0} = k\left( {a_{0} + a_{0\vartheta } } \right) - b_{0\tau }$$

Hence, the parameter set in (53) eventually reads ${\mathbf{p}} = \left( {b_{0\tau } ,\tau_{0} } \right)^{T}$ and $b_{0}$ is then calculated using (54). The LM control parameters are set as in “Initial denominator parameters estimation”.

Table 7 displays the most distinguished results such that two parameters ‘ sets from each of the six pole spectrum families are selected. The corresponding ${\text{Res}}\left( {{\mathbf{p}}_{opt} } \right)$, IAE and ITAE criteria values from unit step responses, and RMS values from Nyquist plots for $\omega_{fin} = 0.1$ and $\omega_{fin} = {1.7}\times{1}0^{{ - {2}}}$ are provided in Table 8.

**Table 7:** HETDM transfer function numerator parameters computed via the LM method.

$G_{HETDM} \left( s \right)$#	$q_{HETDM}$#	$b_{0}$	$b_{0\tau }$	$\tau_{0}$(s)
I-a-1	I-a	1.6119	1.6081	6.7858 × 10^–2^
I-b-1	I-b	31.1794	− 31.1472	2.8797 × 10^–3^
II-a-1	II-a	0.2560	0.2430	8.3566 × 10^–2^
II-d-1	II-d	9.2203	− 9.2156	1.7417 × 10^–3^
III-a-1	III-a	0.1881	0.1345	5.5557 × 10^–2^
III-c-1	III-c	1.6129	1.6077	5.6997 × 10^–2^
IV-a-1	IV-a	7.9628 × 10^–3^	− 1.3623 × 10^–5^	5.0648
IV-b-1	IV-b	1.5266	1.5218	5.2279 × 10^–2^
V-a-1	V-a	1.5158 × 10^–4^	− 1.3623 × 10^–5^	16.8245
V-c-1	V-c	3.2182	5.1884 × 10^–2^	196.7613
VI-c-1	VI-c	3.3697	− 0.1497	3.8464 × 10^–2^
VI-e-1	VI-e	0.1780	0.1440	0.5089

**Table 8:** HETDM time-domain and frequency-domain model errors (ad Table 7).

$G_{HETDM} \left( s \right)$#	${\text{Res}}\left( {{\mathbf{p}}_{opt} } \right)$	IAE	ITAE	RMS ($\times 10^{ - 3}$)$\omega_{fin} = 0.1$	RMS ($\times 10^{ - 3}$)$\omega_{fin} = {1.7}\times{1}0^{{ - {2}}}$
I-a-1	0.2539	259.0	1.610 × 10^5^	2.638	3.272
I-b-1	0.2315	192.6	1.115 × 10^5^	2.738	3.215
II-a-1	0.2682	264.5	1.702 × 10^5^	2.591	3.317
II-d-1	0.2441	253.6	1.559 × 10^5^	2.673	3.234
III-a-1	0.6150	268.0	1.716 × 10^5^	2.620	3.280
III-c-1	0.5899	254.5	1.576 × 10^5^	2.621	3.282
IV-a-1	3.383 × 10^–5^	766.6	3.883 × 10^5^	5.545	9.078
IV-b-1	7.218 × 10^–4^	766.8	3.882 × 10^5^	5.545	9.078
V-a-1	5.643 × 10^–5^	271.1	1.563 × 10^5^	3.887	2.383
V-c-1	7.002 × 10^–11^	268.4	1.523 × 10^5^	3.851	2.371
VI-c-1	4.923 × 10^–3^	274.3	1.577 × 10^5^	3.847	2.420
VI-e-1	4.078 × 10^–4^	266.8	1.496 × 10^5^	3.846	2.419

Compared to SFOTDMs (Tables 2, 3), IAE and ITAE criteria values of unit step responses for HETDMs have been enhanced in all six families of models. Contrariwise, frequency-domain error measures have not been improved; the results in Table 8 are very close to those displayed in Table 3.

**Figure 12:**
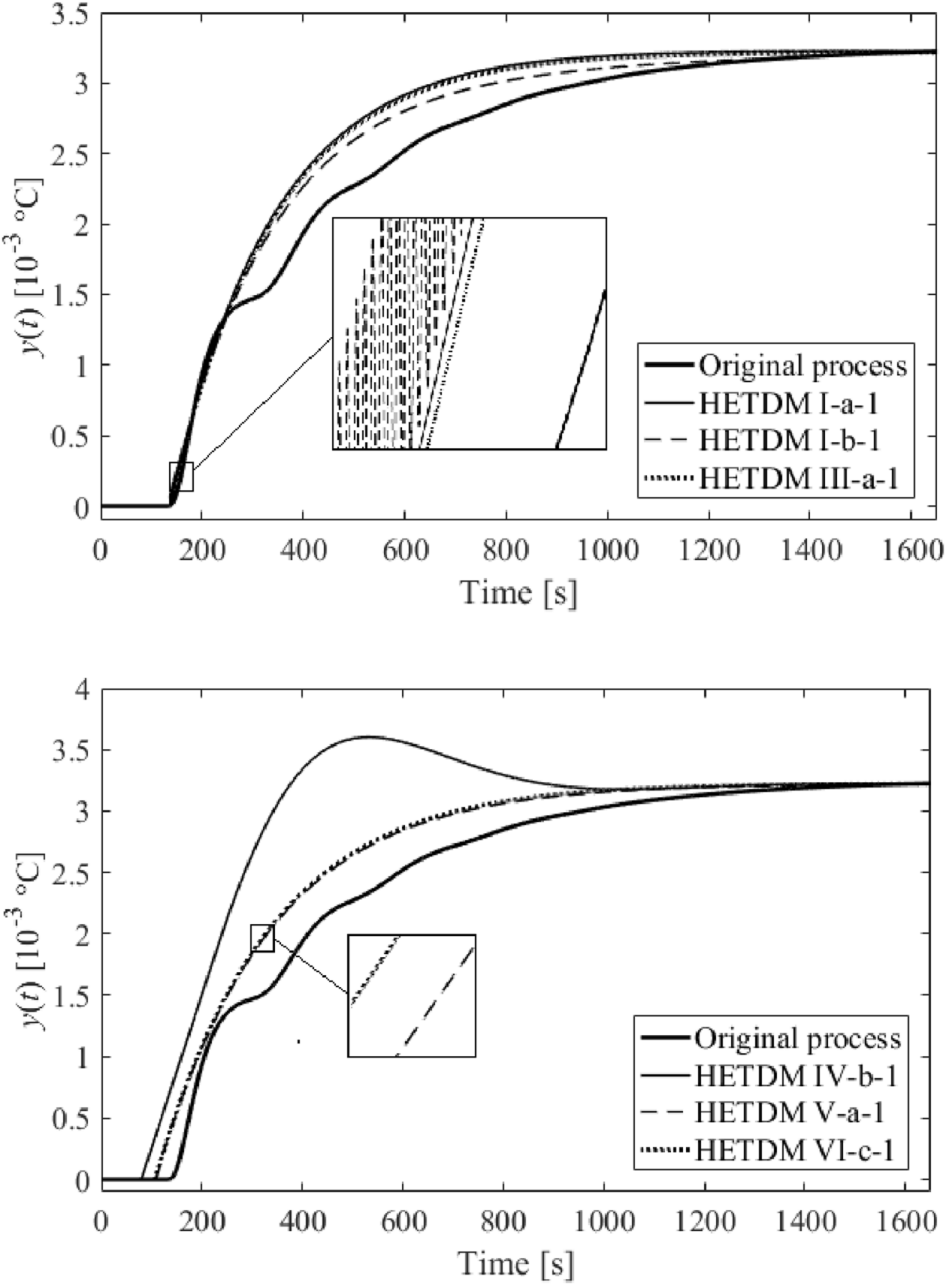
Comparison of selected HETDM unit step responses (vs. the original process response)—LM method used for transfer function numerator parameters computation.

**Figure 13:**
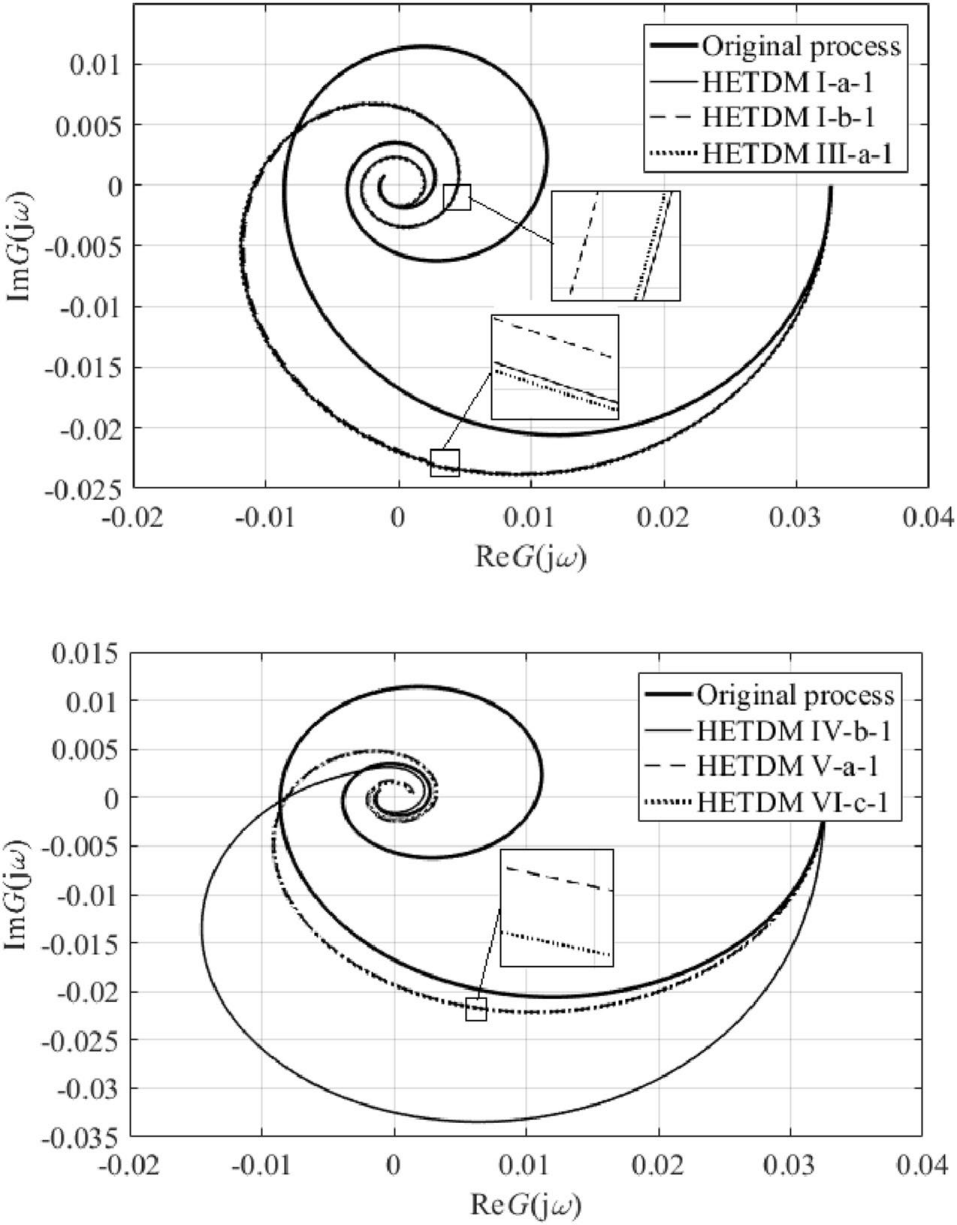
Comparison of selected HETDM Nyquist plots (vs. the original process response)—LM method used for transfer function numerator parameters computation.

The unit step responses and Nyquist plots of some selected HETDMs are displayed in Figs. 12 and 13, respectively. The models are selected such they dynamic responses differ significantly. Regarding non-displayed responses, they are very close to some displayed ones. Namely, in the time domain, II-a-1 almost meets III-a-1, yet the latter is faster. II-d-1 and III-c-1 are close to I-a-1. IV-a-1 and IV-b-1 almost coincide, yet IV-b-1 is faster. The same assertion holds for the pair V-c-1 and V-a-1. Finally, VI-e-1 is nearly identical to V-a-1. In the frequency domain, II-a-1 is close to the pair I-a-1 and III-a-1 at the whole frequency range. II-d-1 approaches I-a-1 at low frequencies and I-b-1 at higher ones. III-c-1 is almost identical to III-a-1 for all frequencies. Finally, pairs V-a-1/V-c-1 and VI-c-1/VI-e-1 have frequency responses very close to each other.

#### Numerator parameters estimation using Nelder–Mead method from single relay test

Now, let us solve the same task as in the preceding subsection via the NM method. The optimization problem is formulated as follows

55 $$\begin{gathered} \left[ {b_{0} ,b_{0\tau } ,\tau_{0} } \right]^{*} = \arg \min f\left( {b_{0} ,b_{0\tau } ,\tau_{0} } \right) \hfill \\ f\left( {b_{0} ,b_{0\tau } ,\tau_{0} } \right): = \,\left( {{\text{Re}} \left. {G_{o,HETDM} \left( s \right)} \right|_{{s = {\text{j}}\omega_{osc} }} + 1} \right)^{2} + \left( {{\text{Im}} \left. {G_{o,HETDM} \left( s \right)} \right|_{{s = {\text{j}}\omega_{osc} }} } \right)^{2} \hfill \\ {\text{s}}{.}\,{\text{t}}{.}: - \tau_{0} < 0 \hfill \\ \end{gathered}$$

where the HETDM characteristic RQPs are fixed as in Table 5 and $\tau = \tau_{s}$. Again, the value of $\omega_{osc}$ is taken from the saturation relay test. Note that the cost function with real and imaginary parts of $G_{o,HETDM} \left( s \right)$ is used in (55) rather than that with the amplitude and phase since numerical experiments give better results (in the sense of (4)).

The most outstanding results are provided in Table 9 (two parameters’ sets from each of the six pole spectrum families are selected again). Table 10 displays corresponding performance measures and implies that the accuracy of HETDMs in Table 9 is very close (or slightly worse) to models obtained in “Numerator parameters estimation using Levenberg–Marquardt method” (except for models from family I). This means that the models give better accuracy than SFOTDMs in the time domain yet only comparable ones in the frequency domain.

**Table 9:** HETDM transfer function numerator parameters computed via the NM method.

$G_{HETDM} \left( s \right)$#	$q_{HETDM}$#	$b_{0}$	$b_{0\tau }$	$\tau_{0}$(s)
I-a-2	I-a	3.3239	− 6.6270 × 10^–2^	34.4855
I-b-2	I-b	3.4070 × 10^–2^	− 1.4979 × 10^–3^	17.9309
II-a-2	II-a	0.6075	− 0.1026	16.8460
II-c-2	II-c	5.4684 × 10^–2^	− 4.4208 × 10^–3^	33.5382
III-c-2	III-c	3.5490	− 0.2907	17.2018
III-f-2	III-f	2.0429 × 10^–4^	− 1.2638 × 10^–5^	33.7698
IV-a-2	IV-a	8.1536 × 10^–3^	− 1.1019 × 10^–4^	72.8193
IV-c-2	IV-c	1.3123 × 10^–4^	− 4.2513 × 10^–6^	44.2441
V-a-2	V-a	1.4396 × 10^–4^	− 4.3970 × 10^–6^	57.5067
V-c-2	V-c	3.3085	− 4.7473 × 10^–2^	86.1898
VI-b-2	VI-b	3.2988	− 4.1318 × 10^–2^	79.1822
VI-d-2	VI-d	3.3020	− 4.4630 × 10^–2^	75.5706

**Figure 14:**
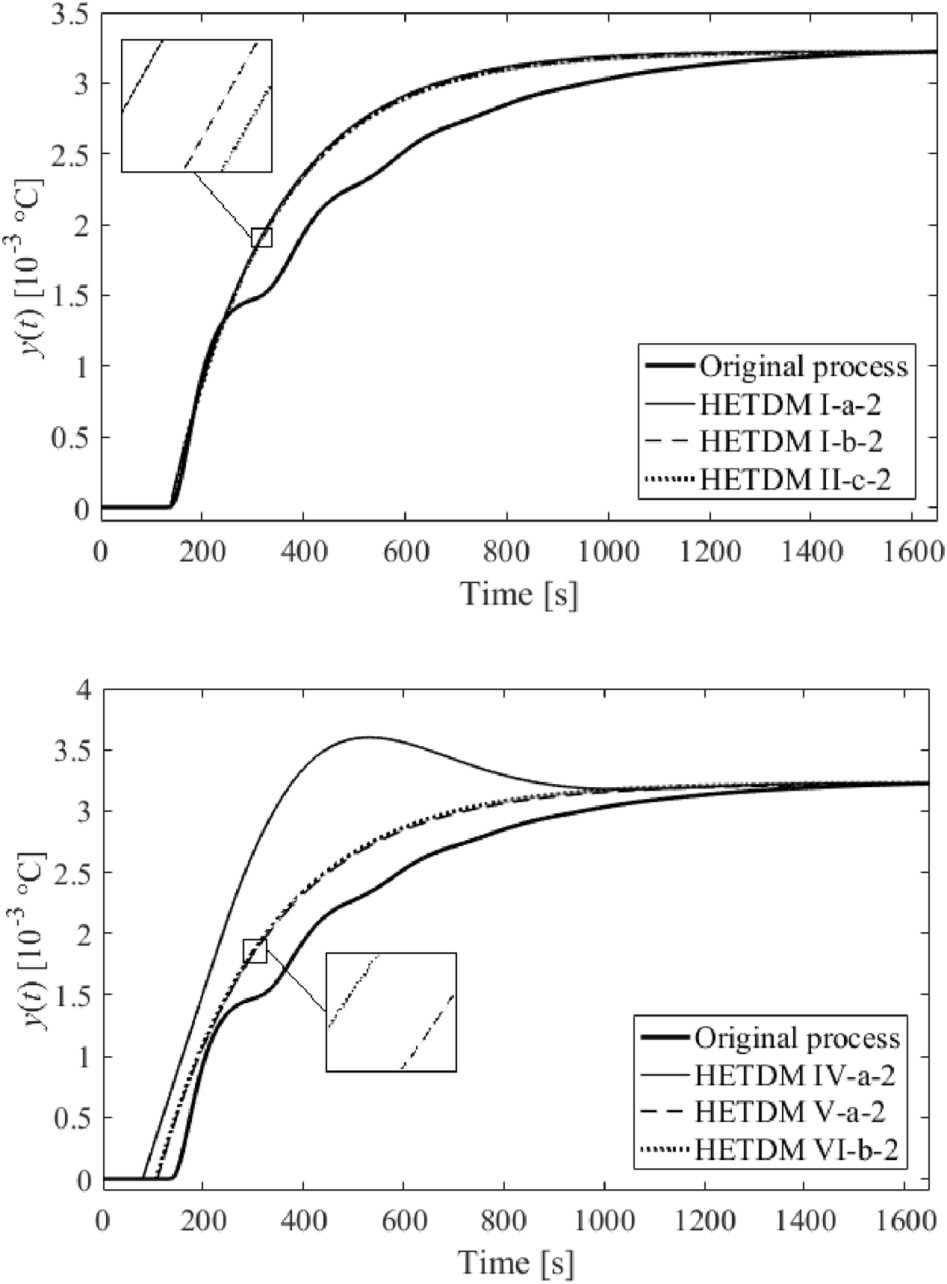
Comparison of selected HETDM unit step responses (vs. the original process response)—NM method used for transfer function numerator parameters computation.

**Figure 15:**
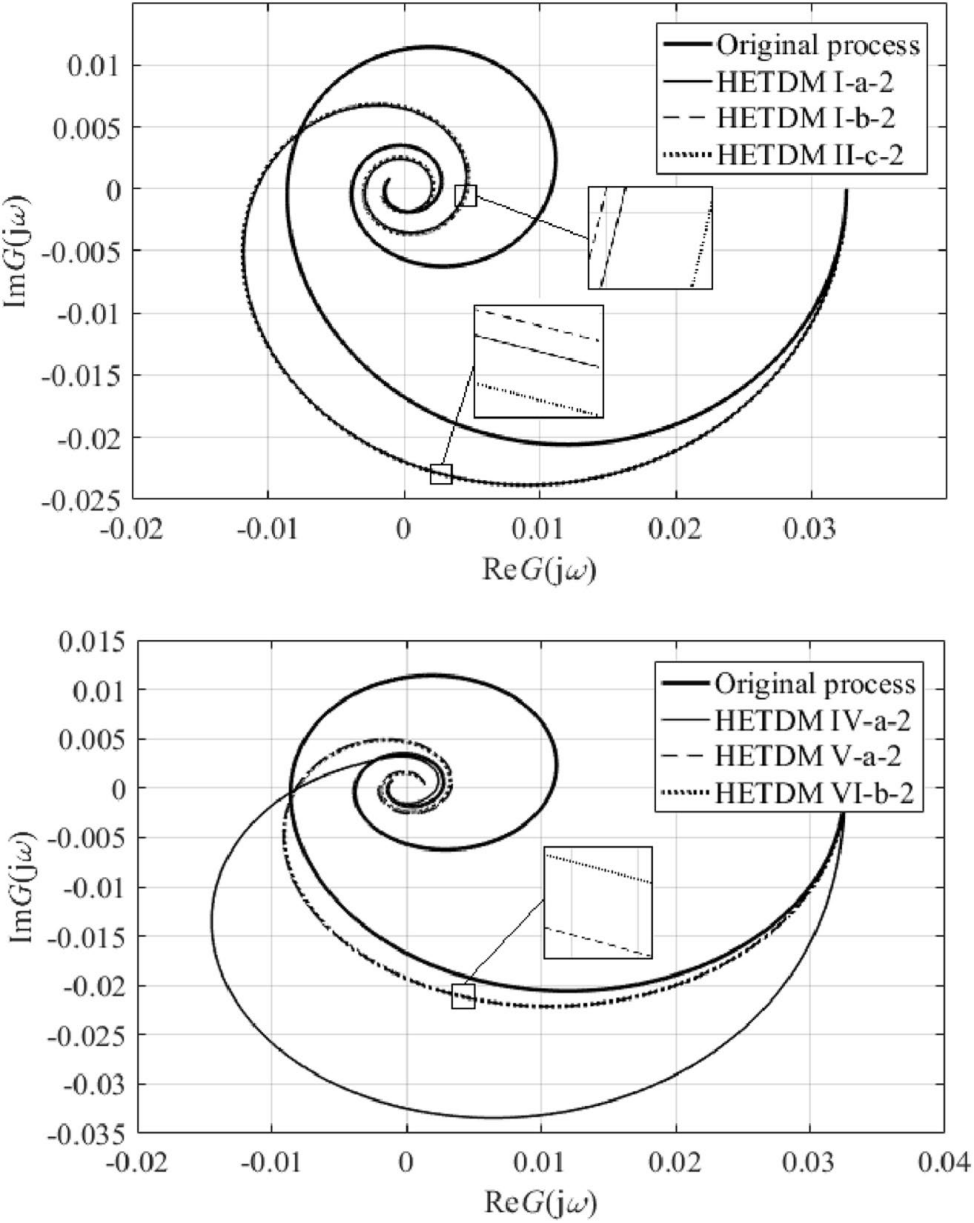
Comparison of selected HETDM Nyquist plots (vs. the original process response)—NM method used for transfer function numerator parameters computation.

Unit step responses and Nyquist plots of selected models are given in Figs. 14 and 15, respectively. Note again that other characteristics are close to some displayed ones. In the time domain, responses for model families I, II, and III almost coincide when I-a-2 is the fastest and II-c-2 is the slowest. The same assertion holds for families V and VI (VI-b-2 and V-a-2 give the fastest and the slowest response, respectively), while models IV significantly differ from the others. Nyquist plots of III-c-2 and III-f-2 are closest to each other at low frequencies and the pair II-c-2/III-f-2 at high ones. The pair VI-b-2/VI-d-2 almost coincides at the whole frequency range. Notice that although perfect optimization based on the measured ultimate data (see Table 10) is reached, model IV-a-2 does not provide excellent frequency response.

**Table 10:** HETDM time-domain and frequency-domain model errors (ad Table 9).

$G_{HETDM} \left( s \right)$#	$f\left( {b_{0}^{*} ,b_{0\tau }^{*} ,\tau_{0}^{*} } \right)$	IAE	ITAE	RMS ($\times 10^{ - 3}$)$\omega_{fin} = 0.1$	RMS ($\times 10^{ - 3}$)$\omega_{fin} = {1.7}\times{1}0^{{ - {2}}}$
I-a-2	1.5926 × 10^–5^	262.9	1.642 × 10^5^	2.641	3.264
I-b-2	1.5721 × 10^–5^	272.1	1.739 × 10^5^	2.659	3.258
II-a-2	1.5841 × 10^–5^	270.4	1.730 × 10^5^	2.643	3.350
II-c-2	1.6675 × 10^–5^	269.2	1.725 × 10^5^	2.575	3.274
III-c-2	1.5701 × 10^–5^	268.1	1.698 × 10^5^	2.656	3.253
III-f-2	1.6830 × 10^–5^	269.8	1.7293 × 10^5^	2.579	3.285
IV-a-2	1.2921 × 10^–8^	769.9	3.890 × 10^5^	5.578	9.121
IV-c-2	4.467 × 10^–22^	766.8	3.884 × 10^5^	5.585	9.100
V-a-2	0	271.6	1.566 × 10^5^	3.852	2.340
V-c-2	1.829 × 10^–8^	271.4	1.533 × 10^5^	3.865	2.440
VI-b-2	1.299 × 10^–8^	273.0	1.539 × 10^5^	3.860	2.461
VI-d-2	1.461 × 10^–8^	272.9	1.537 × 10^5^	3.864	2.462

Figure 15 indicates that there must exist a frequency warping. Indeed, the RMS error value of the Nyquist plot for model V-a-2 and $\omega \in \left[ {0,1.7\times{1}0^{{ - {2}}} } \right]$ is better than that for model VI-b-2. However, the curve for the latter model is closer to the original Nyquist plot than that for the former one. This implies that the model accuracy cannot be judged solely on the shape of characteristics.

Another question is whether optimizing more than three transfer function numerator parameters can improve the HETDMs accuracy.

#### Numerator/denominator parameters estimation using Nelder–Mead method via Autotune Variation Plus experiment

**Figure 16:**
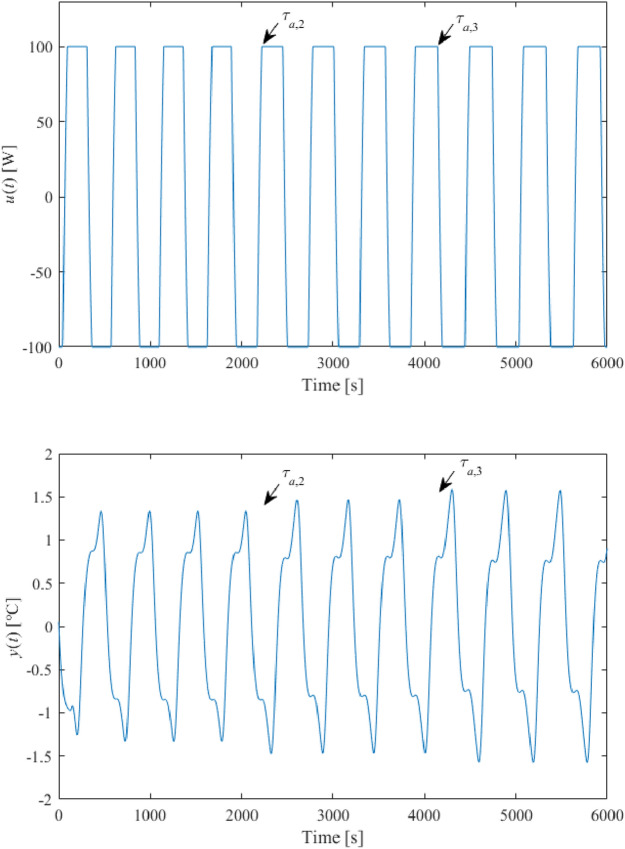
Relay experiment responses (with artificial delays).

By substituting (54) into (45), the 8-parameter model is ready to be identified, i.e., $q_{HETDM}$ is not fixed. We do let use the ATV + technique (see “ATV + technique”) that dictates the use of three artificial delays yielding the estimation of three additional critical points in the frequency domain. Hence, let $\tau_{a,2} = {77}{\text{.021}}$ s as per (9) and take linear values in the neighborhood of this delay as,$\tau_{a,1} = {61}{\text{.617}}$, $\tau_{a,3} = {92}{\text{.425}}$. The common saturated-relay-feedback experiment (see Fig. 16) yields $A_{2} = {1}{\text{.46}}0\,{{^\circ {\text{C}}}}, \, T_{osc,2} = {560}{\text{.03}}\,{\text{s}}$, $A_{1} = {1}{\text{.328}}\,{{^\circ {\text{C}}}}, \, T_{osc,1} = {527}{\text{.23}}\,{\text{s}}$, and $A_{3} = {1}{\text{.570}}\,{{^\circ {\text{C}}}}$, $T_{osc,3} = {591}{\text{.56}}\,{\text{s}}$, respectively. Note that $k_{sat} = 185.1$, $\overline{A} = 0.555$ as in “Simple model parameter estimation using the relay-based experiment”.

The NM method is hence used to solve the problem

56 $$\begin{gathered} {\mathbf{p}}^{*} = \left[ {b_{0} ,b_{0\tau } ,\tau_{0} ,\tau ,a_{2} ,a_{1} ,a_{0} ,a_{0\vartheta } ,\vartheta } \right]^{*} = \arg \min f\left( {b_{0} ,b_{0\tau } ,\tau_{0} ,\tau ,a_{2} ,a_{1} ,a_{0} ,a_{0\vartheta } ,\vartheta } \right) \hfill \\ f\left( {b_{0} ,b_{0\tau } ,\tau_{0} ,\tau ,a_{2} ,a_{1} ,a_{0} ,a_{0\vartheta } ,\vartheta } \right): = \hfill \\ \,\sum\nolimits_{i = 0}^{3} {\left( {\left| {\left. {G_{o,HETDM} \left( s \right)} \right|_{{s = {\text{j}}\omega_{osc,i} }} } \right| - 1} \right)^{2} + \left( {\measuredangle \left. {G_{o,HETDM} \left( s \right)} \right|_{{s = {\text{j}}\omega_{osc,i} }} + \pi - \tau_{a,i} \omega_{osc,i} } \right)^{2} } \hfill \\ {\text{s}}{.}\,{\text{t}}{.}:\left[ { - \tau_{0} , - \tau , - a_{2} , - a_{1} , - \vartheta } \right] < 0 \hfill \\ \end{gathered}$$

where $i = 0$ is associated with $\tau_{a,0} = 0$. Conditional inequalities are incorporated via the barrier function $f_{b} \left( {\tau_{0} ,\tau ,a_{2} ,a_{1} ,\vartheta } \right) = - \sum\nolimits_{{x \in \left\{ {\tau_{0} ,\tau ,a_{2} ,a_{1} ,\vartheta } \right\}}}^{{}} {\ln \left( {1 - {\text{e}}^{ - x} } \right)}$. If any inequality is broken, the model is not stable or feasible. The remaining parameters, however, can be non-positive.

**Figure 17:**
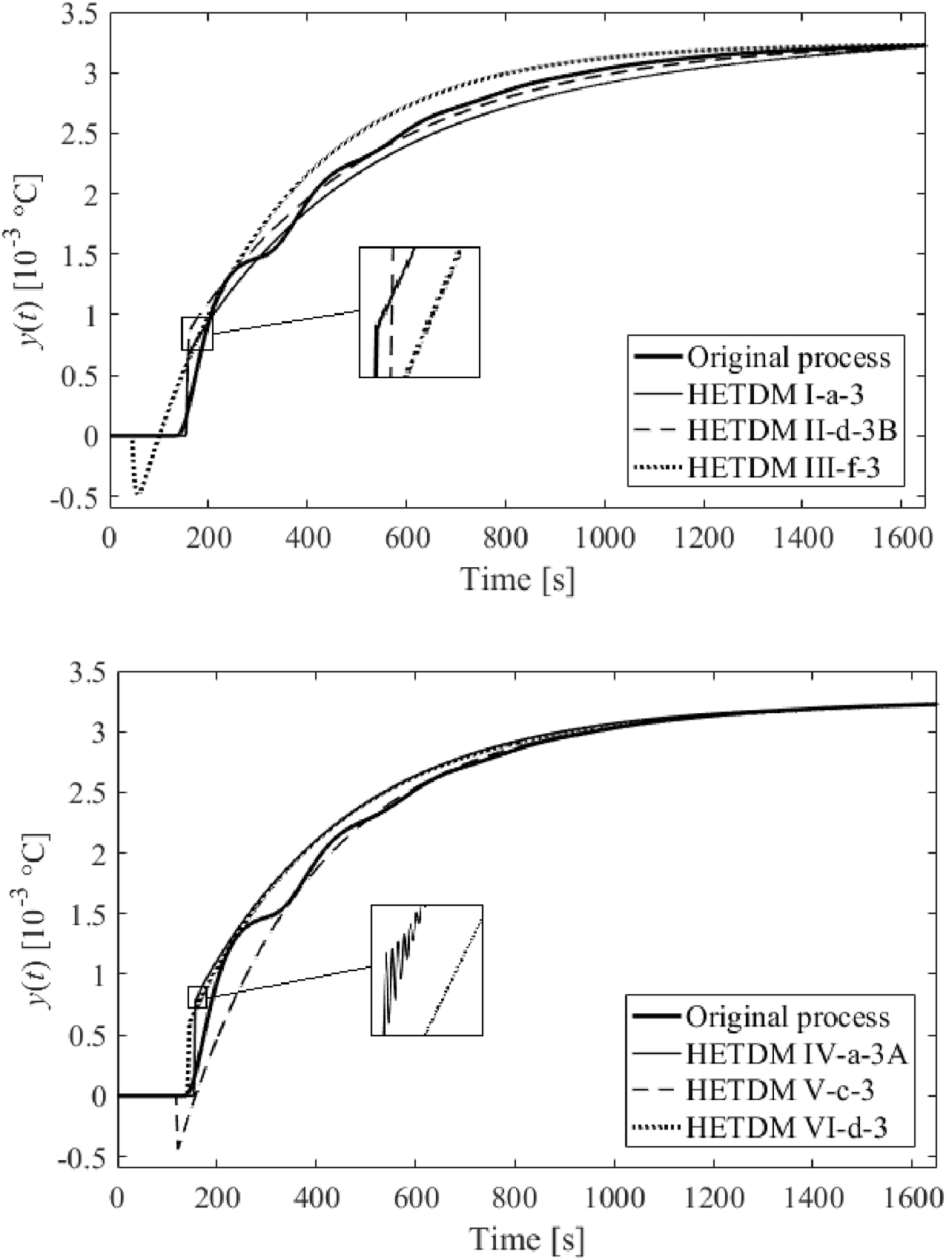
Comparison of selected HETDM unit step responses (vs. the original process response)—NM method used for complete transfer function parameters estimation.

**Figure 18:**
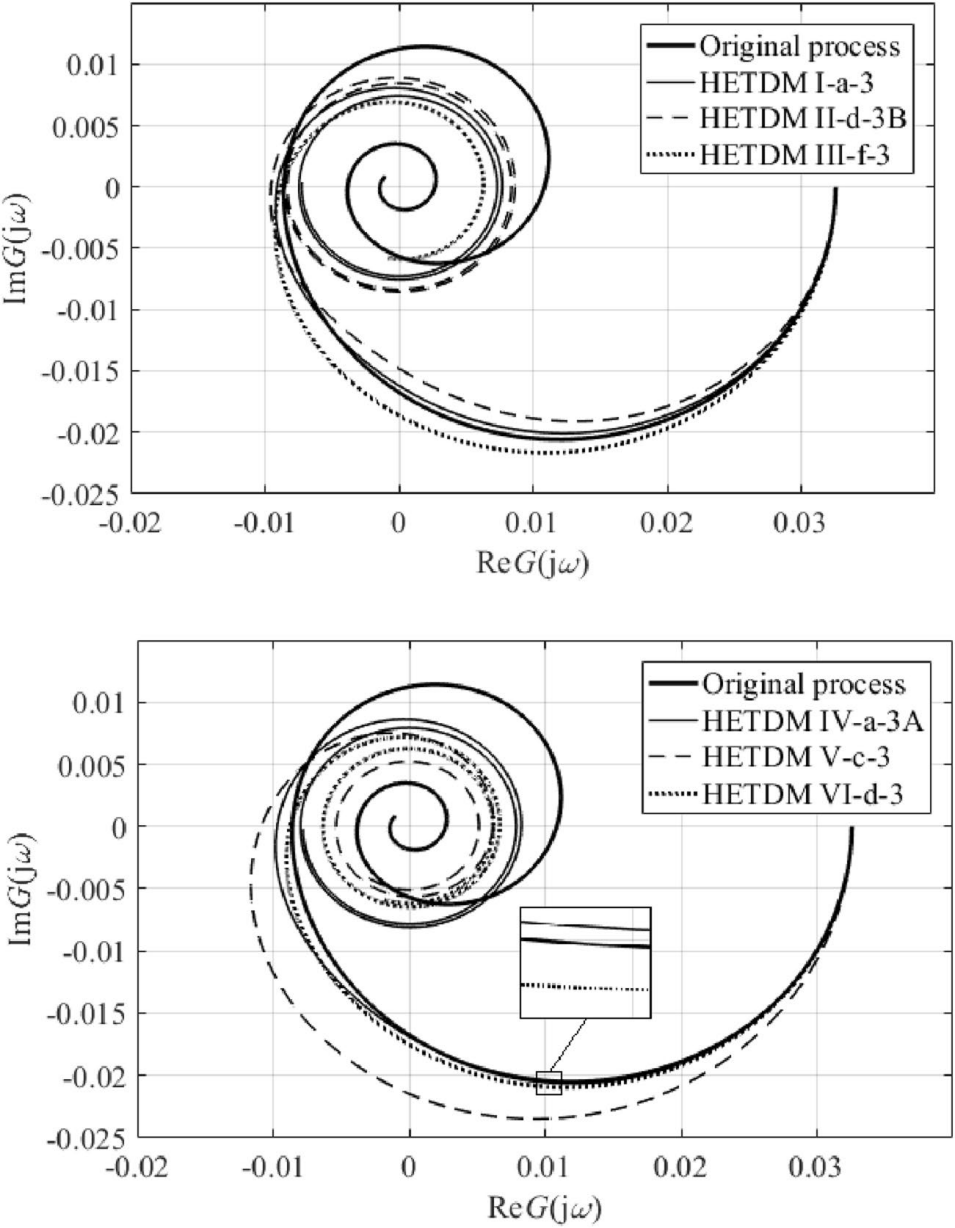
Comparison of selected HETDM Nyquist plots (vs. the original process response)—NM method used for complete transfer function parameters estimation.

The standard setting $\,\,\gamma_{r} = 1$, $\,\,\gamma_{e} = 2$, $\,\,\gamma_{oc} = \gamma_{ic} = \gamma_{s} = 0.5$ is adopted while different values of $\beta$—see (18)—and the initial simplex size are benchmarked. The initial RQP parameter estimates come from Table 5 and let

57 $${}^{1}b_{0} = {}^{1}b_{0\tau } = \frac{{k\left( {{}^{1}a_{0} + {}^{1}a_{0\vartheta } } \right)}}{2},\,{}^{1}\tau_{0} = 0.1\,{\text{s}}$$

and the value of ${}^{1}\tau$ depends on the particular model family, see Table 2, i.e., ${}^{1}\tau = 136.7,\,79.377,\,106.869$, or $106.39$ s. Results are summarized in Table 11 (again, two eventual HETDMs from each family of models are taken). Corresponding performance measures in the time and frequency domains are provided to the reader in Table 12, and particular dynamic responses for selected models are displayed in Figs. 17 and 18.

**Table 11:** HETDM transfer function parameters computed via the NM method (artificial delays are used).

$G_{HETDM} \left( s \right)$#	${\mathbf{p}}^{*} = \left[ {b_{0} ,b_{0\tau } ,\tau_{0} ,\tau ,a_{2} ,a_{1} ,a_{0} ,a_{0\vartheta } ,\vartheta } \right]^{*}$
I-a-3	$$\left[ \begin{gathered} {78}{\text{.6312}}, - 75.0398,3.6561,154.4474,1.5388,4.0237 \times 10^{4} , - 17.1592, \hfill \\ 127.4072,16.3674 \hfill \\ \end{gathered} \right]$$
I-b-3	$$\left[ \begin{gathered} 0.2140, - 0.1949,7.6231,152.0900,1.7902,203.6374, - 0.3557,0.9418, \hfill \\ 3.6365 \hfill \\ \end{gathered} \right]$$
II-d-3A	$$\left[ \begin{gathered} 5.7240 \times 10^{ - 2} , - 5.4688 \times 10^{ - 2} ,3.5244,147.9860,8.9664,30.4500, \hfill \\ - 1.0902 \times 10^{ - 2} ,8.9261 \times 10^{ - 2} ,49.0459 \hfill \\ \end{gathered} \right]$$
II-d-3B	$$\left[ \begin{gathered} {0}{\text{.3070}}, - {0}{\text{.2935}},{4}{\text{.6140}},{154}{\text{.7813}},{132}{\text{.0240}},{169}{\text{.0525}},{3}{\text{.0600}} \times 10^{ - 2} , \hfill \\ {0}{\text{.3820}},{12}{\text{.1124}} \hfill \\ \end{gathered} \right]$$
III-c-3	$$\left[ \begin{gathered} 21.7478,2.2564,16.1816,96.9587,10.4363,1.9455 \times 10^{5} , - 0.1392, \hfill \\ 737.0027,36.9030 \hfill \\ \end{gathered} \right]$$
III-f-3	$$\left[ \begin{gathered} - 5.0126 \times 10^{ - 3} ,5.2499 \times 10^{ - 3} ,2.4783,44.9373,9.5103,2.0698, \hfill \\ 2.3833 \times 10^{ - 4} ,7.0449 \times 10^{ - 3} ,7.0138 \hfill \\ \end{gathered} \right]$$
IV-a-3A	$$\left[ \begin{gathered} 0.2023, - 0.1961,2.4123,154.3649,0.7359,63.8551, - 1.5879 \times 10^{ - 6} , \hfill \\ 0.1920,14.6009 \hfill \\ \end{gathered} \right]$$
IV-a-3B	$$\left[ \begin{gathered} {0}{\text{.2383}}, - {0}{\text{.2250}},{3}{\text{.5597}},{144}{\text{.7439}},{4}{\text{.7862}},{132}{\text{.9075}},{6}{\text{.9025}},{0}{\text{.4097}}, \hfill \\ {20}{\text{.0930}} \hfill \\ \end{gathered} \right]$$
V-b-3	$$\left[ \begin{gathered} {4}{\text{.1201}} \times 10^{ - 3} , - {3}{\text{.7047}} \times 10^{ - 3} ,{7}{\text{.2063}},{143}{\text{.7714}},{3}{\text{.3470}},{4}{\text{.0811}}, \hfill \\ - {3}{\text{.7573}} \times 10^{ - 4} ,{1}{\text{.3127}} \times 10^{ - 2} ,{4}{\text{.7608}} \hfill \\ \end{gathered} \right]$$
V-c-3	$$\left[ \begin{gathered} - {30}{\text{.4525}},{33}{\text{.3415}},{3}{\text{.6503}},{117}{\text{.6783}},{43}{\text{.8899}},{2}{\text{.3948}} \times 10^{4} , - {3}{\text{.4535,}} \hfill \\ {92}{\text{.1380}},{4}{\text{.6977}} \hfill \\ \end{gathered} \right]$$
VI-a-3	$$\left[ \begin{gathered} - {11}{\text{.0842}},{11}{\text{.6608}},{3}{\text{.2299}},{42}{\text{.5439}},{34}{\text{.6891}},{5}{\text{.7461}} \times 10^{3} , - {7}{\text{.8893}} \times 10^{ - 3} {,} \hfill \\ {17}{\text{.7086}} \hfill \\ \end{gathered} \right]$$
VI-d-3	$$\left[ \begin{gathered} {46}{\text{.3542}}, - {42}{\text{.8537}},{4}{\text{.1791}},{140}{\text{.6583}},{4}{\text{.4339}},{3}{\text{.1243}} \times 10^{4} , \hfill \\ - {5}{\text{.0537}} \times 10^{ - 4} {,107}{\text{.4560,7}}{.0553} \hfill \\ \end{gathered} \right]$$

**Table 12:** HETDM time-domain and frequency-domain model errors (ad Table 11).

$G_{HETDM} \left( s \right)$#	$f\left( {{\mathbf{p}}_{{}}^{*} } \right)$	IAE	ITAE	RMS ($\times 10^{ - 3}$)$\omega_{fin} = 0.1$	RMS ($\times 10^{ - 3}$)$\omega_{fin} = 1.7\times{1}0^{{ - {2}}}$
I-a-3	5.625 × 10^–2^	99.0	6.751 × 10^4^	3.863	0.858
I-b-3	4.881 × 10^–2^	92.3	4.862 × 10^4^	4.178	1.179
II-d-3A	4.213 × 10^–2^	100.0	4.918 × 10^4^	4.072	1.276
II-d-3B	6.655 × 10^–2^	61.6	2.999 × 10^4^	4.334	1.351
III-c-3	2.433 × 10^–2^	200.6	9.792 × 10^4^	3.987	2.753
III-f-3	8.420 × 10^–2^	175.3	8.860 × 10^4^	5.634	1.745
IV-a-3A	5.714 × 10^–2^	115.2	5.695 × 10^4^	4.232	1.472
IV-a-3B	3.823 × 10^–2^	135.8	7.277 × 10^4^	4.544	1.404
V-b-3	4.197 × 10^–2^	119.6	5.984 × 10^4^	4.684	1.432
V-c-3	31.185	90.0	3.180 × 10^4^	3.391	3.661
VI-a-3	0.121	113.8	5.308 × 10^4^	5.798	0.801
VI-d-3	3.407 × 10^–2^	98.9	4.592 × 10^4^	4.781	1.694

Apparently, HETDM parameter identification based on the estimation of four Nyquist plot points results in significantly improved time-domain responses compared to 3-parameter optimizations (see “Numerator parameters estimation using Levenberg–Marquardt method” and “Numerator parameters estimation using Nelder–Mead method from single relay test”). Regarding the model accuracy in the frequency domain, substantial enhancement is achieved for low frequencies, which, however, does not hold for the whole frequency range. As can be deduced from Tables 8, 10, and 12, the better RMS value for $\omega_{fin} = 1.7\times{1}0^{{ - {2}}}$ is, the worse value for $\omega_{fin} = 0.1$ is and vice versa (except for model family IV).

In Fig. 17, nearly nonsmooth step response of HETDM I-a-3 can be observed. An oscillatory response with high-frequency modes of nonnegligible amplitude can be seen for HETDM 4-a-3A.

The remaining (non-displayed) dynamic responses have a similar shape to the displayed ones; however, the plots are not as close as the 3-parameter optimizations. An exception appears for III-f-3, V-c-3, and VI-a-3 where nonminimum-phase-like time responses appear, yet the corresponding Nyquist plots do not prove this feature. The significant step response differences come from diverse input–output delays. Regarding Nyquist plots, the closest curves can be observed for pairs I-a-3/I-b-3 and IV-a-3A/V-b-3 at low frequencies. At higher frequencies, responses differ more significantly, especially those for HETDMs III-c-3, III-f-3, and V-c-3 are far from the remaining ones.

To sum up, most of the models obtained by the solution of task (56) based on the (quadruple) relay-feedback ATV + test gives satisfactory results from the identification point of view.

## Discussion

Let us discuss observations made during the entire HETDM identification procedure and also point out some practical issues.

In our experiments, we have supposed that the hysteresis of the on/off relay is negligible, $\varepsilon \approx 0$. However, a suitable nonzero value has to be set in practice due to the measurement noise. Such a setting prevents the relay from switching too frequently, which may cause failures. Another important practical issue is the static gain estimation, according to (6). Whenever an asymmetry ($B_{ + } \ne B_{ - }$) is induced, the output of the feedback system also becomes asymmetrical. The problem is that the original output setpoint then shifts, which may cause an erroneous estimation of the static gain. The setpoint shifting can be caused by the feedback nature of a relay experiment, process nonlinearities, and/or disturbances. If someone is unsure about the static gain, the step response test can be made. It is worth noting that disturbances also induce asymmetry of the ideal on/off relay experiment. In such a case, various methods of restoring symmetry can be applied^50^.

As a DF generally represents a linear approximation of a nonlinear element, it is impossible to estimate the critical (or another frequency) point exactly by nature. More precisely, neither the found frequency $\omega_{osc}$ nor the loci $G_{m} \left( {{\text{j}}\omega_{osc} } \right) = - 1/N\left( \cdot \right)$ meets the particular values of the actual (measured) process frequency response. This implies that even if the solution of (4) is perfect (see, e.g., HETDM V-a-2 in Table 10), the model does not provide sufficient results from the identification point of view. Besides, even if one or more points of the Nyquist curve are estimated well, the remaining course of the plot may vary from the desired loci significantly (see, e.g., HETDM I-a-3 in Fig. 18).

As can be seen from Tables 7, 9, and 11, relatively high ratios $\left| {b_{0} /b_{0\tau } } \right|$, $\left| {a_{0} /a_{0\vartheta } } \right|$, $\left| {a_{0\vartheta } /a_{0} } \right|$, $\left( {\left| {b_{0} } \right| + \left| {b_{0\tau } } \right|} \right)/\left( {b_{0} + b_{0\tau } } \right)$ often occur. This unpleasant feature yields erroneous steady state computation or numerical instability when simulations (i.e., solving differential equations) due to digital representation of values in computer. Therefore, only some eventual models can be used for control system design and its verification.

Displayed step responses indicate that the initial input/output delay estimation $\tau = \tau_{s} = 136.7$ is pretty good. It can be observed that corresponding model families I, II, and III (see Tables 2, 3, 8, and 10) provide slightly better IAE values and significantly lower overall RMS ($\omega_{fin} = 0.1$) compared to models with different values of $\tau$. On the contrary, the better RMS value for low frequencies ($\omega_{fin} = 1.7\times{1}0^{{ - {2}}}$) is, the lower the ITAE value is obtained, which proves the importance of good low-frequency Nyquist plot estimation for the overall time-domain model response.

The eventual SFOTDMs identified using relay feedback experiment were proved to be sufficient for controller design^12^. By matching SFOTDMs dominant pole loci with those of HETDMs and calculating remaining model parameters, performance measures very close to those of SFOTDMs have been obtained in this study. This implies that the eventual HETDMs based on the data from the single relay test (i.e., without artificial delays) can also be used for control tasks. However, the models seem to be insufficient from the identification point of view. Fortunately, the use of the ATV + experiment has brought about much improved models. As many diverse results have been computed with more or less the same cost function values, the identification problem for HETDMS seems to be multimodal task. Therefore, none of the eventual models (see Table 11) approaches the true parameter values (46) given by physical analysis of the thermal process.

The relay-based experiment can be improved in several ways. For instance, one has to be more careful when setting $k_{sat}$ of the saturated relay (see Fig. 4). The ideal setting should result in sinusoidal-like sustained oscillations of $u\left( t \right)$. However, Figs. 9 and 16 indicate that $k_{sat}$ was set too high. On the other hand, one has to be aware of the necessary existence of sustained oscillations. Another way how to enhance the coefficient value estimation is to capture multiple points of the Nyquist plot, which may yield better matching of process and model curves for a wide frequency range^47–49^.

## Conclusions

This study should have examined whether it is reasonable to identify parameters of a complex model of a thermal circuit system with internal delays by a parameter identification of a simpler delayed model followed by the models’ poles matching. As the identification tool, the standard on/off relay with biased and unbiased feedback test and the relay with saturation have been used. The latter relay should have yielded a more accurate estimation of points on the frequency curve corresponding to sustained oscillations data. Once the simple model is found under a single feedback experiment, its dominant pole loci (of an infinite spectrum) are matched to those of the complex model, giving rise to the characteristic quasi-polynomial coefficients. A simple graphical-based method has been analytically derived to find these loci. The Levenberg–Marquardt method has been applied to solve the pole assignment task. Surprisingly, although only a few poles have been prescribed, some other uncontrolled poles have also been matched. Based on the single-test data, the remaining model parameters have been estimated by the solution of a nonlinear optimization problem (using the Nelder–Mead vs. the Levenberg–Marquardt methods). It has been proved that both the models have had similar time and frequency domain performances. While the eventual models may be sufficient from the control point of view, they fail regarding the accuracy of identified parameters. On the other hand, the proposed procedure enables the estimation of multiple parameters under a single relay test, which is its main benefit. However, we have also performed the ATV + test with artificial delays to get multiple relay-feedback data, which has resulted in much better eventual models.

In the Discussion section, we have touched on some issues that have to be considered in practice and proposed possible further improvements to the proposed concept. Besides, one may apply advanced and more sophisticated optimization approaches to solve the tasks raised in this study. For instance, metaheuristic methods can be benchmarked in the future^98^. In addition, real-life experiments will be made to prove the concept.

## Data Availability

Data are available by L.P. upon request.
